# PRiSM project: e-Delphi study on the role of specialist palliative care services in the care of people living beyond cancer

**DOI:** 10.1177/02692163251376957

**Published:** 2025-10-22

**Authors:** Amy Taylor, Andrew Davies, PRiSM e-Delphi Group

**Affiliations:** 1 School of Medicine, Trinity College Dublin, Dublin, Ireland; 2 Academic Department of Palliative Medicine, Our Lady’s Hospice & Care Services, Dublin, Ireland; 3 School of Medicine, University College Dublin, Dublin, Ireland

**Keywords:** Palliative care, cancer survivors, survivorship, Delphi technique

## Abstract

**Background:**

Specialist palliative care provides holistic care for individuals with health-related suffering and those close to them. Historically this included people with advanced cancer, however, people living beyond cancer also have significant unmet needs. Specialist palliative care could potentially provide support, but evidence for their role is limited.

**Aim:**

To generate consensus opinion among European experts about the role of specialist palliative care for people living beyond cancer.

**Design:**

The PRiSM project (Specialist Palliative care’s Role in Cancer Survivorship Model) was an electronic Delphi study. Participants provided anonymous feedback on statements across three rounds. Consensus was pre-defined as ⩾75% within each group. Spearman’s Rank Order Correlation determined stability and the Chi square test for goodness of fit compared group outcomes.

**Setting/participants:**

European professional experts and patient advocates were invited via email, forming two groups undertaking simultaneous processes: specialist palliative care (86 experts representing 19 countries/regions) and oncology (54 experts representing 17 countries/regions).

**Results:**

Eighty specialist palliative care and 49 oncology experts completed the process. Outcomes were achieved about the general core functions of specialist palliative care and areas requiring education, and their potential role for people living beyond cancer. Sixty statements reached stable consensus in both groups. Comparing other outcomes, 16 statements reached consensus in only one group, nine with a significant difference.

**Conclusions:**

European specialist palliative care and oncology experts reached consensus that specialist palliative care should have a role in supporting people living beyond cancer, specifically in pain management. Results will inform a new care model.

**What is already known about the topic?**

- Specialist palliative care services provide holistic care for individuals with health-related suffering and those close to them, which historically included people with advanced cancer.
- Many people living beyond cancer also have significant unmet needs across physical, psychological, social and informational domains.
- There is potential alignment between the unmet needs of people living beyond cancer and specialist palliative care services, but evidence is limited.

**What this paper adds?**

- Core functions of specialist palliative care services should include management of acute and chronic pain and other physical problems related to cancer and its treatment.
- Specialist palliative care services should contribute to a multidisciplinary team supporting people with a history of cancer and no evidence of disease, specifically in supporting pain management.
- A number of barriers exist, including limited resources, education and misconceptions about the nature of specialist palliative care.

**Implications for practice, theory or policy**

- Improving the care of people living beyond cancer will require major changes in healthcare policy, funding, service provision and training of healthcare professionals.
- The consensus statements generated by this study will be used to inform development of a new model of care for the role of specialist palliative care services for people who have or had cancer, to be evaluated in future research.

## Introduction

The European Commission estimates that there are over 12 million people with a history of cancer in Europe, with this number expected to increase by 24% over the next decade.^1^ This rise is attributed to the growing and ageing population,^2,3^ as well as developments in anticancer treatment and supportive care services, enabling people to live longer ‘with, through and beyond cancer’.^4^ (p. 1) As a result, the ongoing care of these individuals has become a major healthcare (and health economic) issue.

Cancer survivorship refers to the unique physical, psychological, social and economic issues affecting individuals with a history of cancer.^5^ The essential components of survivorship care, as specified in the relevant Institute of Medicine report,^6^ include prevention (of late effects and cancer), surveillance (for late effects and cancer), intervention (for cancer and cancer treatment sequelae) and coordination (between oncology and primary care services). The report (and hence cancer survivorship) relates to ‘the period following first diagnosis and treatment and prior to the development of a recurrence. . .or death’.^6^ (p. 23) These individuals with no evidence of disease who have completed anticancer treatment can be referred to as long-term cancer survivors, or people ‘living beyond cancer’.^4^ (p. 6)

Specialist palliative care services are specialist teams that provide holistic care for individuals with health-related suffering and those close to them.^7^ Traditionally, specialist palliative care services have been involved in the care of people with advanced/progressive cancer, although they are increasingly involved in the care of people earlier in the cancer trajectory, including those receiving anticancer therapies (early palliative care).^8^ However, many people living beyond cancer also have significant unmet needs across physical, psychosocial, financial and informational domains.^9,10^ These health-related problems may relate to the cancer, cancer treatment and/or another chronic disease and encompass a range of issues, such as pain, sexual dysfunction, psychological distress, fear of cancer recurrence, financial and occupational problems and educational needs.^11^ Previous articles have suggested that a palliative care (i.e. holistic) approach should at least be applied to these individuals,^12,13^ however, other articles support actual involvement of specialist palliative care services, ranging from targeted interventions,^13,14^ to the provision of more wide-ranging support including care coordination and advance care planning.^15,16^ In particular, it has been suggested that specialist palliative care services should have a role in managing people with chronic cancer treatment-related pain (and/or problems with opioid abuse), given the relative expertise of specialist palliative care in managing cancer-related pain, including opioid prescribing.^17–19^ However, there is great diversity in specialist palliative care teams regarding membership, services and availability,^20^ and a paucity of evidence (with studies limited in number and quality) to support the suggestions about their potential role for people living beyond cancer.^12,21^

The PRiSM Project (Specialist Palliative Care's Role in Cancer Survivorship Model) aims to develop European expert consensus about the role of specialist palliative care services in cancer survivorship. Specifically, this article addresses the definition of palliative care/specialist palliative care, the general core functions of specialist palliative care services and the potential role of specialist palliative care services for people living beyond cancer (and the perceived barriers to greater integration from specialist palliative care services).

## Methods

### Design

**Figure 1.**
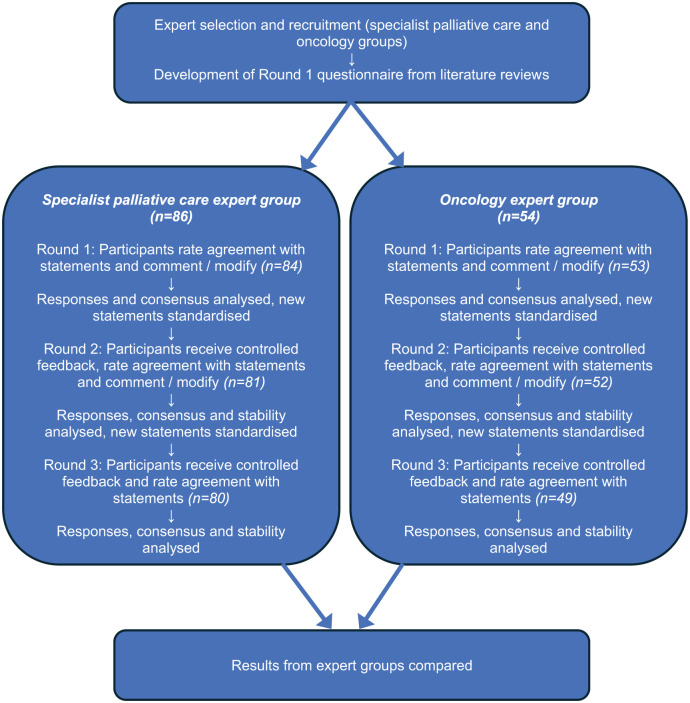
Flow chart showing PRiSM e-Delphi process.

The PRiSM project was an electronic Delphi (e-Delphi) study where participants provided anonymous feedback on relevant statements over three rounds with the aim to obtain (or not) stable consensus about the individual statements (Figure 1).^22,23^ The study employed a modified Delphi technique since Round 1 incorporated 105 questions/statements developed from a review of the medical literature^23^ relating to cancer survivorship terminology, and the role of specialist palliative care services for people living beyond cancer.^21^ The statements were developed by the authors of the review, with feedback from members of an academic department of palliative medicine in Ireland with experience of Delphi methodology. The study is reported according to the Guidance on Conducting and REporting DElphi Studies (CREDES) in palliative care.^22^

### Setting

Members of a specialist palliative care multidisciplinary team from Ireland were selected to take part in a pilot of Round 1 on the Welphi^®^ online platform (Lisbon, Portugal),^24^ leading to adjustments for usability. Following this, official study rounds commenced and concurrent e-Delphi processes were undertaken with two separate European expert groups related to specialist palliative care and oncology between April and June 2024 utilising the Welphi^®^ platform.^24^

### Population

Criteria for expert selection included individuals with leadership roles in relevant professional and patient advocacy organisations (and associated national organisations/member societies), individuals highlighted from a background literature review on the role of specialist palliative care services in cancer survivorship,^21^ individuals with European expertise in palliative care and/or oncology in clinical and/or academic and/or other relevant settings, particularly cancer survivorship, and individuals recommended by those meeting previous criteria. Participants were excluded if they did not reside in a European country or have adequate English language proficiency to complete the written informed consent and e-Delphi processes.

### Sampling

Purposive sampling was used. Potential participants were identified by contacting relevant European palliative care/oncology organisations (i.e. professional associations, patient advocacy groups) and requesting nominations. The first or corresponding author of relevant articles from the study’s background literature review^21^ and individuals with relevant expertise within the inclusion criteria across geographical areas were also targeted. Other participants were identified through snowball sampling (i.e. suggested by those who entered the study).

### Recruitment

Potential participants were sent an invitation email. Those who responded were provided with a Participant Information Leaflet outlining the study design, commitment and rationale, invited to ask questions and asked to return a signed study-specific Consent Form.

### Data collection

Baseline information was collected about participant age, sex, country of residence and profession if relevant. Participants also confirmed their suggested group allocation (specialist palliative care or oncology). Once entered into the platform, participant identification and responses were anonymised from the researchers. To begin each round, participants were emailed personalised links via Welphi^®24^ to enter one of two simultaneous identical Delphi processes.

In each round, participants were asked to rate their level of agreement with the statements using a 5-point Likert scale (i.e. strongly disagree, moderately disagree, neither agree nor disagree, moderately agree and strongly agree), and to suggest any modifications to the statements. They were also asked to provide free-text responses to open-ended questions, and general comments about the topic/e-Delphi process.

Rounds 2 and 3 included both original statements and new/modified statements (see Data Analysis and Results Sections), and participants received controlled feedback, that is anonymised results of their group from the previous round.^25,26^ Round 3 did not include those statements with stable consensus after rounds 1 and 2.

Each round was open for about 2 weeks. Reminder emails were sent to non-responders via the platform towards the deadline to encourage participation, and all participants were notified about round closure. The process was terminated after three rounds, since stable consensus had been achieved for the majority of statements.^27^

### Data analysis

Qualitative content analysis of free text responses and comments was undertaken by two researchers (AT and AD) independently then collaboratively to determine additions/alterations to the statements between rounds.

Descriptive statistics were primarily utilised to report the e-Delphi process results, for example frequency of various ratings of agreement with individual statements. Consensus was pre-defined as ⩾75% having either moderate/strong agreement, moderate/strong disagreement or neutrality.^28,29^ Stability of the statement ratings across two rounds was determined using Spearman’s Rank Order Correlation (rho).^27^ Values of 0.80–1.00 represent a very strong association, 0.50–0.80 a moderate association, 0.20–0.5 a fair association and <0.2 a poor association.^30^ The Chi square (χ^2^) test for goodness of fit compared agreement between the specialist palliative care and oncology groups.^31^ Statistical tests were carried out using Statistical Package for the Social Sciences (SPSS) version 29.0.2.0.

### Ethical issues

Ethical approval was received from Trinity College Dublin Faculty of Health Sciences Research Ethics Committee (project number 2786). The study was registered on the Comet Initiative database (https://www.comet-initiative.org/Studies/Details/3097). All data was treated as confidential.

## Results

Eighty-six specialist palliative care experts representing 19 European countries/regions agreed to take part in the study, with 84 (98%) completing round 1, 81 (94%) completing round 2 and 80 (93%) completing round 3. Equally, 54 oncology experts representing 17 European countries agreed to take part in the study, with 53 (98%) completing round 1, 52 (96%) completing round 2 and 49 (91%) completing round 3. Table 1 shows the participants’ characteristics.

**Table 1.** Characteristics of expert groups.

Characteristic	Specialist palliative care expert group (*n = 86*)	Oncology expert group (*n = 54*)
Sex *n* (%)	Female *45* (52) Male *41* (48)	Female *31* (57) Male *23* (42)
Age (years)	Median = 52 (range = 33–68)	Median = 50 (range = 31–70)
Country/region of residence *n* (%)	Austria *3* (3) Belgium *1* (1) Denmark *6* (7) France *5* (6) Germany *6* (7) Greece *1* (1) Ireland *15* (17) Italy *3* (3) Malta *2* (3) Netherlands *2* (3) Norway *5* (6) Poland *6* (7) Portugal *4* (5) Romania *2* (3) Serbia *1* (1) Spain *5* (6) Sweden *3* (3) Switzerland *3* (3) United Kingdom *13* (15)	Belgium *1* (2) Cyprus *1* (2) Denmark *1* (2) France *4* (7) Germany *1* (2) Ireland *6* (11) Italy *6* (11) Netherlands *6* (11) Norway *3* (6) Poland *1* (2) Portugal *4* (7) Serbia *1* (2) Slovenia *1* (2) Spain *2* (4) Sweden *2* (4) Switzerland *3* (6) United Kingdom *11* (19)
Discipline *n* (%)	Healthcare professional – *82* (95) - physician *59* (69) - nurse *16* (19) - pharmacist *2* (2) - dietitian *1* (1) - medical manager *1* (1) - medical scientist *1* (1) - occupational therapist *1* (1) - psychologist *1* (1) Non-healthcare professional – *4* (5) - Public and Patient Involvement group members *4* (5)	Healthcare professional – *49* (91) - physician *36* (66) - nurse *8* (15) - physiotherapist *2* (4) - pharmacist *1* (2) - psychologist *1* (2) - skin therapist *1* (2) Non-healthcare professional – *5* (9) - cancer patient advocacy group members *5* (9)

Note: *n*=number of participants (italicised).

During round 1, the specialist palliative care participants made 527 comments, and the oncology participants made 243 comments. These comments resulted in 15 of the original statements being excluded, 20 of the original statements being modified for clarity and 89 new statements being formulated for round 2. During round 2, the specialist palliative care participants made a further 182 comments, and the oncology participants made a further 105 comments. These comments resulted in eight statements being modified for clarity for round 3. There were no new statements in round 3, and 56 statements were not included as they had already achieved (stable) consensus.

Regarding the statements relating to the definition of palliative care/specialist palliative care, the core functions of specialist palliative care services and the potential role and barriers for specialist palliative care services for people living beyond cancer, outcomes were achieved for 107 final statements. Consensus was achieved for 71 of these statements in the specialist palliative care group, and for 65 in the oncology group (Tables 2–4; Supplemental Tables S1 and S2). Sixty statements reached consensus in both groups. There was a significant difference in 9 of the 16 statements that reached consensus in only one group (supplemental Table S3).

**Table 2.** Consensus statements on core functions of specialist palliative care.

Statement reaching consensus	% specialist palliative care group	% oncology group	Agreement between groups
Expert group agrees that the following are ‘core functions’ of specialist palliative care services			
Management of pain	97	98	χ^2^ = 0.082; *p* = *0.775*
Management of other physical symptoms	97	96	χ^2^ = 0.353; *p* = *0.553*
Management of psychological problems	97	96	χ^2^ = 0.353; *p* = *0.553*
Emotional support (patient)	96	98	χ^2^ = 0.489; *p* = *0.484*
Emotional support (family)	95	96	χ^2^ = 0.144; *p* = *0.704*
Spiritual support	83	**72**	χ^2^ = 4.484; ***p*** = ***0.034***
Social assistance	82	**61**	χ^2^ = 15.684; ***p*** ***<*** ***0.001***
Co-ordination of care	89	96	χ^2^ = 2.883; *p* = *0.090*
Advance care planning	97	98	χ^2^ = 0.489; *p* = *0.484*
End-of-life care	96	100	N/A
Bereavement care	92	81	χ^2^ = 7.109; ***p*** = ***0.008***
Research	97	93	χ^2^ = 0.726; *p* = *0.394*
Education (other healthcare professionals)	99	100	N/A
Expert group agrees that specialist palliative care services should manage the following types of pain			
Acute cancer-related pain^a^	93	94	χ^2^ = 0.234; *p* = *0.629*
Acute cancer treatment-related pain^a^	85	92	χ^2^ = 2.211; *p* = *0.137*
Chronic cancer-related pain^b^	93	96	χ^2^ = 1.017; *p* *=* *0.313*
Chronic cancer treatment-related pain^b^	86	96	χ^2^ = 4.355; ***p*** = ***0.037***
Chronic cancer/cancer treatment-related pain – in collaboration with the multidisciplinary chronic pain team	97	96	χ^2^ = 0.503; *p* = *0.478*
Individuals with cancer/cancer treatment–related pain and a previous history of opioid misuse/abuse/addiction	94	98	χ^2^ = 1.503; *p* = *0.220*
Individuals with cancer/cancer treatment–related pain and ongoing problems of opioid misuse/abuse/addiction	91	96	χ^2^ = 1.907; *p* = *0.167*
Individuals with chronic cancer/cancer treatment–related pain and a previous history of opioid misuse/abuse/addiction – in collaboration with the multidisciplinary chronic pain team	89	90	χ^2^ = 0.058; *p* = *0.810*
Individuals with chronic cancer/cancer treatment–related pain and ongoing problems of opioid misuse/abuse/addiction – in collaboration with the multidisciplinary chronic pain team	89	94	χ^2^ = 1.306; *p* = *0.253*
Individuals with cancer/cancer treatment–related pain and a previous history of opioid misuse/abuse/addiction – in collaboration with addiction services	93	94	χ^2^ = 0.134; *p* = *0.714*
Individuals with cancer/cancer treatment–related pain and ongoing problems of opioid misuse/abuse/addiction – in collaboration with addiction services	91	96	χ^2^ = 1.355; *p* = *0.244*
Expert group agrees that specialist palliative care services should manage the following types of non-pain symptoms/physical problems			
Acute cancer-related symptoms/physical problems*	94	87	χ^2^ = 4.475; ***p*** = ***0.034***
Acute cancer treatment-related symptoms/physical problems^a^	87	79	χ^2^ = 2.309; *p* = *0.129*
Acute symptoms/physical problems related to non-cancer causes^a^	77	**74**	χ^2^ = 0.251; *p* = *0.617*
Chronic cancer-related symptoms/physical problems^b^	95	96	χ^2^ = 0.144; *p* = *0.704*
Chronic cancer treatment-related symptoms/physical problems^b^	88	93	χ^2^ = 1.110; *p* = *0.292*
Chronic symptoms/physical problems related to non-cancer causes^b^	**65**	81	χ^2^ = 6.611; ***p*** = ***0.010***

Key: % group bold = subthreshold 75% that is consensus was not reached in that expert group; *p* values italicised; *p* value bold = Chi square (χ^2^) test for goodness of fit and one degree of freedom comparing agreement between groups was significant at a level of ⩽0.05.

a Duration <3 months^32^. ^b^Duration ⩾3 months^32^.

**Table 3.** Consensus statements about current knowledge and skills of specialist palliative care services and areas requiring further education/training.

Statement reaching consensus	% specialist palliative care group	% oncology group	Agreement between groups
Expert group agrees that specialist palliative care services generally have the knowledge and skills to manage the following			
Acute cancer-related pain^a^	99	94	χ^2^ = 8.894; ***p*** = ***0.003***
Acute cancer treatment-related pain^a^	95	94	χ^2^ = 0.066; *p* = *0.798*
Acute pain related to non-cancer causes^a^	**61**	83	χ^2^ =10.193; ***p*** = ***0.001***
Chronic cancer-related pain^b^	100	94	N/A
Chronic cancer treatment-related pain^b^	86	96	χ^2^ = 4.355; ***p*** = ***0.037***
Chronic pain related to non-cancer causes	**62**	78	χ^2^ = 5.499; ***p*** = ***0.019***
Individuals with cancer/cancer treatment–related pain and a previous history of opioid misuse/abuse/addiction	85	93	χ^2^ = 3.029; *p* = *0.082*
Individuals with cancer/cancer treatment–related pain and ongoing problems of opioid misuse/abuse/addiction	78	94	χ^2^ = 7.537; ***p*** = ***0.006***
Acute cancer-related symptoms/physical problems^a^	98	87	χ^2^ = 24.929; ***p*** ***<*** ***0.001***
Acute cancer treatment-related symptoms/physical problems^a^	94	97	χ^2^ = 19.306; ***p*** ***<*** ***0.001***
Acute symptoms/physical problems related to non-cancer causes^a^	78	**72**	χ^2^ = 1.143; *p* = *0.285*
Chronic cancer-related symptoms/physical problems^b^	95	96	χ^2^ = 0.066; *p* = *0.798*
Chronic cancer treatment-related symptoms/physical problems^b^	89	86	χ^2^ = 0.239; *p* = *0.625*
Regarding pain management, expert group agrees that specialist palliative care services should have more education and/or appropriate training in the following areas			
Interventional pain techniques	92	91	χ^2^ = 0.240; *p* = *0.877*
Physiotherapy interventions	93	78	χ^2^ = 15.784; ***p*** ***<*** ***0.001***
Occupational therapy interventions	82	**74**	χ^2^ = 2.768; *p* = *0.096*
Transcutaneous electrical nerve stimulation, scrambler therapy	80	**73**	χ^2^ = 1.306; *p* = *0.253*
Psychological and behavioural therapies/interventions	92	90	χ^2^ = 0.122; *p* = *0.727*
Pharmacological interventions in addiction recovery	84	75	χ^2^ = 2.420; *p* = *0.120*
Regarding management of non-pain symptoms/physical problems, expert group agrees that specialist palliative care services should have more training in the following areas			
Assessment of acute side effects of anticancer treatment (especially novel anticancer treatments)	99	77	χ^2^ = 171.000; ***p*** ***<*** ***0.001***
Management of acute side effects of anticancer treatment (especially novel anticancer treatments)	96	84	χ^2^ = 21.066; ***p*** ***<*** ***0.001***
Assessment of chronic/long-term effects of anticancer treatment (especially novel anticancer treatments)	99	85	χ^2^ = 64.474; ***p*** ***<*** ***0.001***
Management of chronic/long-term effects of anticancer treatment (especially novel anticancer treatments)	97	86	χ^2^ = 14.763; ***p*** ***<*** ***0.001***
Assessment of late/delayed effects of anticancer treatment (especially novel anticancer treatments)	99	79	χ^2^ = 139.592; ***p*** ***<*** ***0.001***
Management of late/delayed effects of anticancer treatment (especially novel anticancer treatments)	96	82	χ^2^ = 28.487; ***p*** ***<*** ***0.001***

Key: % group bold = subthreshold 75% that is consensus was not reached in that expert group; *p* values italicised; *p* value bold = Chi square (χ^2^) test for goodness of fit and one degree of freedom comparing agreement between groups was significant at a level of ⩽0.05.

a Duration < 3 months^32^. ^b^Duration ⩾3 months.^32^

**Table 4.** Consensus statements on the extended role of specialist palliative care services for individuals with a history of cancer who have completed anticancer treatment and have no evidence of disease.

Statement reaching consensus	% specialist palliative care group	% oncology group	Agreement between groups
Expert group agrees that specialist palliative care services should have a role in supporting the following components of care			
Management of pain	76	81	χ^2^ = 1.150; *p* = *0.284*
Management of other physical symptoms/problems	**73**	75	χ^2^ = 0.223; *p* *=* *0.637*
Expert group disagrees that specialist palliative care services should have a role in supporting the following components of care			
Prevention of second cancers	89	87	χ^2^ = 9.577; ***p*** = ***0.002***
Surveillance for recurrence or second cancers	88	90	χ^2^ = 0.825; *p* = *0.364*
Managing financial problems (‘financial toxicity’)	79	**68**	χ^2^ = 11.367; ***p*** ***<*** ***0.001***
Expert group agrees that specialist palliative care services generally have the knowledge and skills to support the following components of care			
Management of pain	88	92	χ^2^ = 0.843; *p* = *0.359*
Management of other physical symptoms/problems	86	84	χ^2^ = 0.266; *p* = *0.606*
Management of psychological distress	83	81	χ^2^ = 0.134; *p* = *0.715*
Supporting caregivers	78	**69**	χ^2^ = 1.849; *p* = *0.174*
Expert group disagrees that specialist palliative care services generally have the knowledge and skills to support the following components of care			
Prevention of second cancers	90	92	N/A
Surveillance for recurrence or second cancers	86	88	χ^2^ = 4.185; ***p*** = ***0.041***
Managing financial problems (‘financial toxicity’)	76	**47**	χ^2^ = 0.047; *p* = *0.829*
Expert group agrees that the following are barriers to extending the input of specialist palliative care services to this group			
Financial resources	82	**71**	χ^2^ = 4.027; ***p*** = ***0.045***
Human resources	90	81	χ^2^ = 5.388; ***p*** ***<*** ***0.020***
Time resources	85	85	χ^2^ = 0.019; *p* = *0.892*
Lack of relevant education and training	81	**62**	χ^2^ = 11.770; ***p*** ***<*** ***0.001***
Misperceptions about the nature of palliative care	76	75	χ^2^ = 0.038; *p* = *0.846*
Type of service			
Expert group agrees that			
A dedicated multidisciplinary specialist team is required	89	87	χ^2^ = 0.045; *p* = *0.832*
Specialist palliative care services should be an extended member of this team that is support with relevant problems/issues	74	88	χ^2^ = 4.978; ***p*** = ***0.026***
Expert group disagrees that			
Specialist palliative care services should not contribute to this team	80	83	χ^2^ = 0.002; *p* = *0.962*
Specialist palliative care services should lead this team	76	80	χ^2^ = 0.058; *p* = *0.810*

Key: % group bold = subthreshold 75% that is consensus was not reached in that expert group; *p* values italicised; *p* value bold = Chi square (χ^2^) test for goodness of fit and one degree of freedom comparing agreement between groups was significant at a level of ⩽0.05.

Responses for all consensus statements demonstrated stability between rounds. In the specialist palliative care group consensus statements, responses showed very strong association (rho = 0.80–1.0) for 16 statements, moderate association (rho = 0.50–0.79) for 53 statements and fair association (rho = 0.2–0.49) for 2 statements. In the oncology group consensus statements, responses between rounds demonstrated very strong association (rho = 0.80–1.0) for 28 statements, moderate association (rho = 0.50–0.79) for 35 statements and fair association (rho = 0.2–0.49) for 2 statements.

### Definitions of palliative care and specialist palliative care

The majority of experts (63.1% specialist palliative care group and 69.8% oncology group) favoured the World Health Organization’s definition of palliative care, that is ‘palliative care is an approach that improves the quality of life of patients (adults and children) and their families who are facing problems associated with life-threatening illness. It prevents and relieves suffering through the early identification, correct assessment and treatment of pain and other problems, whether physical, psychosocial or spiritual’.^33^ Equally, the majority of experts (82.1% specialist palliative care group and 81.1% oncology group) selected the European Association of Palliative Care’s description as their preferred definition of specialist palliative care, that is ‘Specialist palliative care is provided by specialised services for patients with complex problems not adequately covered by other treatment options. Specialist palliative care services require a team approach, combining a multi-professional team with an interdisciplinary mode of work. Team members must be highly qualified and should have their main focus of work in palliative care’.^34^ ^(p. 686)^

### Core functions of specialist palliative care services

Tables 2 and 3 show the statements regarding general core functions, current specialist palliative care knowledge and skills and areas requiring further education/training that reached consensus in both groups (*n* = 46), the specialist palliative care group only (*n* = 6) and the oncology group only (*n* = 3).

### Extended role of specialist palliative care

Table 4 shows the statements regarding the extended role of specialist palliative care services for individuals with a history of cancer who have completed anticancer treatment and have no evidence of disease (i.e. people living beyond cancer) that reached consensus in both groups (*n* = 14), the specialist palliative care group only (*n* = 5) and the oncology group only (*n* = 2).

## Discussion

### Main findings

This e-Delphi process has generated important information about the potential role of specialist palliative care services for people living beyond cancer,^4^ and will be utilised in the development of a European-focussed model of care. The e-Delphi reiterated the core functions of specialist palliative care services, and there was good concordance between the two expert groups. The only exceptions were the provision of spiritual support, and the provision of social assistance (both reached consensus in the specialist palliative care group but not the oncology group). Importantly, there is much overlap between the core functions of specialist palliative care services and the unmet needs of individuals living beyond cancer,^9,10^ and statements on the specific role of specialist palliative care services have been defined.

### What this study adds?

In terms of chronic cancer-related/cancer treatment-related pains, there was not only consensus that this was a core function of specialist palliative care services, but that these services already had the knowledge and skills to undertake this role (in collaboration with chronic pain teams). Furthermore, this role should include the patients with a previous history, or ongoing problems, with opioid abuse/addiction (in collaboration with addiction services). Importantly, the two expert groups specifically agreed that specialist palliative care services should be involved in management of chronic pain in individuals living beyond cancer, which aligns with previous suggestions.^35–39^ Moreover, there was similar consensus for chronic cancer-related/cancer treatment-related non-pain symptoms.

The management of chronic pain in individuals living beyond cancer is fundamentally different from the approach used in people with advanced cancer (i.e. typical palliative care patients)^40^ and the specialist palliative care group especially identified the need for further education/training in non-pharmacological interventions (e.g. psychological/behavioural techniques and transcutaneous electrical nerve stimulation/scrambler therapy), interventional techniques and pharmacological interventions for the treatment of addiction. Similarly, they identified the need for further education/training in the assessment/management of long term, and delayed, effects of cancer treatments. Importantly, the specialist palliative care group disagreed that they had the knowledge and skills to support other essential components of survivorship care, such as coordination of care, surveillance for recurrence or second cancers and prevention of second cancers.^6^ Furthermore, the specialist palliative care group disagreed that dealing with financial toxicity was within their remit (or ability).

Both groups of experts agreed that there needed to be a dedicated multidisciplinary team to support individuals living beyond cancer, and that while specialist palliative care services should contribute to this team, they should not lead this team. The ideal team is likely a broad group of many specialties, including but not restricted to oncology and primary care. Importantly, the team should include persons with expertise in non-specialist palliative care core functions (e.g. cancer prevention and financial toxicity). However, significant barriers were identified to specialist palliative care services supporting this cohort of people, including misconceptions about palliative care, limited financial resources and limited human (and time) resources. In addition, the need for further education/training was also seen as a major barrier. Specialist palliative care services could only extend care to non-traditional groups like long-term cancer survivors without compromising care to traditional groups if these barriers are overcome.

Consensus was not reached on a number of issues relating to the care of individuals living beyond cancer, including (within the specialist palliative care group) surveillance of psychological distress, managing fear of cancer recurrence, rehabilitation, supporting occupational issues/return to work, supporting caregivers and development of survivorship care plans (Supplemental Tables S1b and S2b). Consensus is typically not reached for all items in a Delphi study,^41^ and differences in opinion may be attributed to variations in personal values, personal experience and exposure to the issues,^42^ as well as questionnaire structure and presentation (and whether written in participant’s native language).^43^ There are no guidelines for when consensus is not achieved,^44^ but face-to-face meetings may be a useful additional procedure (where necessary).

### Strengths and limitations of the study

The strengths of this study are the robust methodology,^26,44^ including the opportunity for participant responses to limit potential researcher biases, and promote alternate/contradictory suggestions.^45,46^ The expert groups were quite large, and quite diverse in nature (countries, disciplines and experience), and uniquely included representation from patient advocacy and Public and Patient Involvement groups. The high response/completion rate (over 90%) improves the project’s credibility, especially since attrition is often high in e-Delphi studies.^26^ However, the panels were skewed towards western European countries, which may have influenced the results. Additionally, a Delphi study’s constructivist nature is inherently limiting,^22^ with findings representing one group at one time,^42^ so additional research should build upon these findings.

This study also does not deal with the roles of oncology and primary care practitioners in cancer survivorship, which will both differ and overlap to some extent with the suggested complementary role of specialist palliative care services. However, given the expanding need for prevention and surveillance (through primary care, and to an extent oncology) and anticancer treatment delivery (through oncology), these services cannot absorb the increasing burden of cancer-related unmet needs, especially as complexity also grows. Collaboration and coordination between services is essential.

## Conclusion

The ongoing care of cancer survivors has become a major healthcare (and health economic) issue in Europe and beyond. Currently, many individuals living beyond cancer have unmet needs, and improving this situation will require major changes in healthcare policy, service funding, service provision (including novel models of care) and training of healthcare professionals. Importantly, services need to be tailored to the relevant population, and take into consideration the locally available resources (and their training/experience). This e-Delphi process will inform these changes, and while the process was undertaken in Europe, the findings are likely to be relevant in other jurisdictions.

## Supplemental Material

sj-docx-1-pmj-10.1177_02692163251376957 – Supplemental material for PRiSM project: e-Delphi study on the role of specialist palliative care services in the care of people living beyond cancer Supplemental material, sj-docx-1-pmj-10.1177_02692163251376957 for PRiSM project: e-Delphi study on the role of specialist palliative care services in the care of people living beyond cancer by Amy Taylor and Andrew Davies in Palliative Medicine
