# Supplementary material for: PRiSM project: e-Delphi study on the role of specialist palliative care services in the care of people living beyond cancer
Source: Palliat Med. 2025 Oct 22;40(1):50–61. doi: 10.1177/02692163251376957 (PMC12779773; doi:10.1177/02692163251376957)
Supplement: sj-docx-1-pmj-10.1177_02692163251376957 – Supplemental material for PRiSM project: e-Delphi study on the role of specialist palliative care services in the care of people living beyond cancer [file sj-docx-1-pmj-10.1177_02692163251376957.docx]

**Supplementary materials**

Table of Contents

[Supplementary Tables S1a-b: Specialist palliative care (SPC) expert group results 2](#_Toc195731254)

[S1a. Definition and core functions of Specialist Palliative Care (SPC) – SPC group results 2](#_Toc195731255)

[S1b. Extended role of Specialist Palliative Care for individuals with a history of cancer who have completed anticancer treatment and have no evidence of disease – SPC group results 14](#_Toc195731256)

[Supplementary Tables S2a-b: Oncology (Onc) expert group results 22](#_Toc195731257)

[S2a Definition and core functions of Specialist Palliative Care (SPC) – ONC group results 22](#_Toc195731258)

[S2b. Extended role of Specialist Palliative Care for individuals with a history of cancer who have completed anticancer treatment and have no evidence of disease – ONC group results 32](#_Toc195731259)

[Supplementary Table S3: Comparison of levels of agreement between SPC and ONC groups regarding final statements 40](#_Toc195731260)

# Supplementary Tables S1a-b: Specialist palliative care (SPC) expert group results

S1a. Definition and core functions of Specialist Palliative Care (SPC) – SPC group results

| **Statement** | **Round 1** | | | | | **Round 2** | | | | | | **Round 3** | | | | | | **rho** | **p value** |
| --- | --- | --- | --- | --- | --- | --- | --- | --- | --- | --- | --- | --- | --- | --- | --- | --- | --- | --- | --- |
|  | **SD** | **MD** | **N** | **MA** | **SA** | **SD** | | **MD** | **N** | **MA** | **SA** | **SD** | | **MD** | **N** | **MA** | **SA** |  |  |
| **Core functions of SPCS** | | | | | | | | | | | | | | | | | | | |
| ﻿Management of pain | 0% | 0% | 0% | 5% | 95% | 2% | | 0% | 0% | 1% | 96% |  | | | | | | 0.250* | 0.025 |
|  | 0% | | 0% | 100% | | 2% | | | 0% | 97% | |  |  |  |  |  |  |  |  |
| Management of other physical symptoms | 0% | 0% | 0% | 8% | 92% | 2% | | 0% | 0% | 2% | 95% |  |  |  |  |  |  | 0.325** | 0.003 |
|  | 0% | | 0% | 100% | | 2% | | | 0% | 97% | |  |  |  |  |  |  |  |  |
| ﻿﻿Management of psychological problems | 0% | 0% | 2% | 24% | 74% | 2% | | 0% | 0% | 17% | 80% |  |  |  |  |  |  | 0.658** | <0.001 |
|  | 0% | | 2% | 98% | | 2% | | | 0% | 97% | |  |  |  |  |  |  |  |  |
| Emotional support (patient) | 0% | 0% | 0% | 18% | 82% | 2% | | 1% | 0% | 11% | 85% |  |  |  |  |  |  | 0.586** | <0.001 |
|  | 0% | | 0% | 100% | | 3% | | | 0% | 96% | |  |  |  |  |  |  |  |  |
| Emotional support (family) | 0% | 1% | 2% | 27% | 69% | 2% | | 1% | 1% | 16% | 79% |  |  |  |  |  |  | 0.620** | <0.001 |
|  | 1% | | 2% | 96% | | 3% | | | 1% | 95% | |  |  |  |  |  |  |  |  |
| Spiritual support | 2% | 1% | 12% | 20% | 64% | 4% | | 2% | 11% | 17% | 66% |  |  |  |  |  |  | 0.764** | <0.001 |
|  | 3% | | 12% | 84% | | 6% | | | 11% | 83% | |  |  |  |  |  |  |  |  |
| Social assistance | 1% | 2% | 12% | 36% | 49% | 2% | | 4% | 12% | 35% | 47% |  |  |  |  |  |  | 0.745** | <0.001 |
|  | 3% | | 12% | 85% | | 6% | | | 12% | 82% | |  |  |  |  |  |  |  |  |
| Co-ordination of care | 0% | 1% | 7% | 33% | 58% | 1% | | 2% | 7% | 30% | 59% |  |  |  |  |  |  | 0.766** | <0.001 |
|  | 1% | | 7% | 91% | | 3% | | | 7% | 89% | |  |  |  |  |  |  |  |  |
| Advance care planning | 0% | 2% | 1% | 23% | 74% | 2% | | 0% | 1% | 19% | 78% |  |  |  |  |  |  | 0.646** | <0.001 |
|  | 2% | | 1% | 97% | | 2% | | | 1% | 97% | |  |  |  |  |  |  |  |  |
| End-of-life care | 0% | 0% | 1% | 14% | 85% | 2% | | 0% | 1% | 10% | 86% |  |  |  |  |  |  | 0.599** | <0.001 |
|  | 0% | | 1% | 99% | | 2% | | | 1% | 96% | |  |  |  |  |  |  |  |  |
| Bereavement care | 1% | 1% | 6% | 48% | 44% | 2% | | 0% | 6% | 51% | 41% |  |  |  |  |  |  | 0.819** | <0.001 |
|  | 2% | | 6% | 92% | | 2% | | | 6% | 92% | |  |  |  |  |  |  |  |  |
| Rehabilitation | 1% | 8% | 19% | 42% | 30% | 2% | | 7% | 19% | 44% | 27% |  |  |  |  |  |  | 0.855** | <0.001 |
|  | 9% | | 19% | 72% | | 9% | | | 19% | 71% | |  |  |  |  |  |  |  |  |
| Religious support |  | | | | | 11% | | 17% | 35% | 27% | 10% | 10% | | 16% | 34% | 35% | 5% | 0.732 | <0.001 |
|  |  |  |  |  |  | 28% | | | 35% | 37% | | 26% | | | 34% | 40% | |  |  |
| Research |  |  |  |  |  | 1% | | 2% | 5% | 35% | 57% | 0% | | 3% | 1% | 24% | 73% | 0.65 | <0.001 |
|  |  |  |  |  |  | 3% | | | 5% | 92% | | 3% | | | 1% | 97% | |  |  |
| Education (other healthcare professionals) |  |  |  |  |  | 2% | 0% | | 2% | 20% | 75% | 0% | 0% | | 1% | 9% | 90% | 0.577 | <0.001 |
|  |  |  |  |  |  | 2% | | | 2% | 95% | | 0% | | | 1% | 99% | |  |  |
| **SPCS should manage the following types of pain:** | | | | | | | | | | | | | | | | | | | |
| Acute cancer-related pain (pain for < 3 months) | 0% | 4% | 8% | 20% | 68% | 1% | | 0% | 6% | 20% | 73% |  | | | | | | 0.720** | <0.001 |
|  | 4% | | 8% | 88% | | 1% | | | 6% | 93% | |  |  |  |  |  |  |  |  |
| Acute cancer treatment-related pain (pain for < 3 months) | 1% | 7% | 11% | 32% | 49% | 2% | | 1% | 11% | 22% | 63% |  |  |  |  |  |  | 0.696** | <0.001 |
|  | 8% | | 11% | 81% | | 3% | | | 11% | 85% | |  |  |  |  |  |  |  |  |
| Acute pain related to non-cancer causes (pain for < 3 months) | 11% | 13% | 23% | 21% | 32% | 11% | | 12% | 22% | 25% | 30% |  |  |  |  |  |  | 0.846** | <0.001 |
|  | 24% | | 23% | 53% | | 23% | | | 23% | 55% | |  |  |  |  |  |  |  |  |
| Chronic cancer-related pain (pain for≥3 months) | 2% | 1% | 5% | 26% | 65% | 1% | | 1% | 5% | 21% | 72% |  |  |  |  |  |  | 0.687** | <0.001 |
|  | 3% | | 5% | 91% | | 2% | | | 5% | 93% | |  |  |  |  |  |  |  |  |
| Chronic cancer treatment-related pain (pain for≥3 months) | 2% | 4% | 8% | 32% | 54% | 2% | | 2% | 9% | 28% | 58% |  |  |  |  |  |  | 0.760** | <0.001 |
|  | 6% | | 8% | 86% | | 4% | | | 9% | 86% | |  |  |  |  |  |  |  |  |
| Chronic pain related to non-cancer causes (pain for≥3 months) | 18% | 14% | 11% | 21% | 36% | 21% | | 11% | 9% | 26% | 33% |  |  |  |  |  |  | 0.864** | <0.001 |
|  | 32% | | 11% | 57% | | 33% | | | 9% | 59% | |  |  |  |  |  |  |  |  |
| Individuals with pain and a previous history of opioid misuse / abuse / addiction | 6% | 12% | 15% | 26% | 40% |  | | | | | |  |  |  |  |  |  |  | |
|  | 18% | | 15% | 66% | |  |  |  |  |  |  |  |  |  |  |  |  |  |  |
| Individuals with pain and ongoing problems of opioid misuse / abuse / addiction | 5% | 13% | 20% | 19% | 43% |  |  |  |  |  |  |  |  |  |  |  |  |  |  |
|  | 18% | | 20% | 62% | |  |  |  |  |  |  |  |  |  |  |  |  |  |  |
| Chronic cancer / cancer treatment-related pain – in collaboration with the multidisciplinary chronic pain team |  | | | | | 2% | | 0% | 6% | 38% | 54% | 0% | | 0% | 3% | 31% | 66% | 0.659 | <0.001 |
|  |  |  |  |  |  | 2% | | | 6% | 92% | | 0% | | | 3% | 97% | |  |  |
| Individuals with cancer / cancer treatment–related pain and a previous history of opioid misuse / abuse / addiction |  |  |  |  |  | 1% | | 1% | 11% | 42% | 44% | 1% | | 0% | 5% | 38% | 56% | 0.739 | <0.001 |
|  |  |  |  |  |  | 2% | | | 11% | 86% | | 1% | | | 5% | 94% | |  |  |
| Individuals with cancer / cancer treatment–related pain and ongoing problems of opioid misuse / abuse / addiction |  |  |  |  |  | 1% | | 5% | 15% | 42% | 44% | 1% | | 0% | 9% | 53% | 38% | 0.719 | <0.001 |
|  |  |  |  |  |  | 6% | | | 15% | 76% | | 1% | | | 9% | 91% | |  |  |
| Individuals with chronic cancer / cancer treatment–related pain and a previous history of opioid misuse / abuse / addiction – in collaboration with the multidisciplinary chronic pain team |  |  |  |  |  | 2% | | 1% | 10% | 37% | 49% | 1% | | 0% | 10% | 29% | 60% | 0.768 | <0.001 |
|  |  |  |  |  |  | 3% | | | 10% | 86% | | 1% | | | 10% | 89% | |  |  |
| Individuals with chronic cancer / cancer treatment–related pain and ongoing problems of opioid misuse / abuse / addiction – in collaboration with the multidisciplinary chronic pain team |  |  |  |  |  | 2% | | 2% | 9% | 42% | 44% | 1% | | 0% | 10% | 30% | 59% | 0.782 | <0.001 |
|  |  |  |  |  |  | 4% | | | 9% | 86% | | 1% | | | 10% | 89% | |  |  |
| Individuals with cancer / cancer treatment–related pain and a previous history of opioid misuse / abuse / addiction – in collaboration with addiction services |  | | | | | 4% | | 1% | 10% | 38% | 47% | 1% | | 0% | 6% | 25% | 68% | 0.579 | <0.001 |
|  |  |  |  |  |  | 5% | | | 10% | 85% | | 1% | | | 6% | 93% | |  |  |
| Individuals with cancer / cancer treatment–related pain and ongoing problems of opioid misuse / abuse / addiction – in collaboration with addiction services |  |  |  |  |  | 2% | | 4% | 10% | 27% | 58% | 1% | | 1% | 6% | 15% | 76% | 0.634 | <0.001 |
|  |  |  |  |  |  | 6% | | | 10% | 85% | | 2% | | | 6% | 91% | |  |  |
| **SPCS generally have the knowledge and skills to manage the following:** | | | | | | | | | | | | | | | | | | | |
| Acute cancer-related pain (pain for < 3 months) | 0% | 0% | 5% | 12% | 83% | 0% | | 0% | 1% | 11% | 88% |  | | | | | | 0.743** | <0.001 |
|  | 0% | | 5% | 95% | | 0% | | | 1% | 99% | |  |  |  |  |  |  |  |  |
| Acute cancer treatment-related pain (pain for < 3 months) | 0% | 2% | 8% | 27% | 62% | 0% | | 1% | 4% | 25% | 70% |  |  |  |  |  |  | 0.700** | <0.001 |
|  | 2% | | 8% | 89% | | 1% | | | 4% | 95% | |  |  |  |  |  |  |  |  |
| Acute pain related to non-cancer causes (pain for < 3 months) | 6% | 13% | 23% | 23% | 36% | 5% | | 10% | 23% | 23% | 38% |  |  |  |  |  |  | 0.857** | <0.001 |
|  | 19% | | 23% | 59% | | 15% | | | 24% | 61% | |  |  |  |  |  |  |  |  |
| Chronic cancer-related pain (pain for≥3 months) | 2% | 0% | 1% | 24% | 73% | 0% | | 0% | 0% | 21% | 79% |  |  |  |  |  |  | 0.662** | <0.001 |
|  | 2% | | 1% | 97% | | 0% | | | 0% | 100% | |  |  |  |  |  |  |  |  |
| Chronic cancer treatment-related pain (pain for≥3 months) | 4% | 5% | 8% | 36% | 48% | 1% | | 4% | 9% | 32% | 54% |  |  |  |  |  |  | 0.868** | <0.001 |
|  | 9% | | 8% | 84% | | 5% | | | 9% | 86% | |  |  |  |  |  |  |  |  |
| Chronic pain related to non-cancer causes (pain for≥3 months) | 11% | 13% | 19% | 25% | 32% | 9% | | 15% | 15% | 27% | 35% |  |  |  |  |  |  | 0.902** | <0.001 |
|  | 24% | | 19% | 57% | | 24% | | | 15% | 62% | |  |  |  |  |  |  |  |  |
| Individuals with pain and a previous history of opioid misuse / abuse / addiction | 6% | 14% | 21% | 37% | 21% |  | | | | | |  |  |  |  |  |  |  | |
|  | 20% | | 21% | 58% | |  |  |  |  |  |  |  |  |  |  |  |  |  |  |
| Individuals with pain and ongoing problems of opioid misuse / abuse / addiction | 6% | 18% | 23% | 33% | 20% |  |  |  |  |  |  |  |  |  |  |  |  |  |  |
|  | 24% | | 23% | 53% | |  |  |  |  |  |  |  |  |  |  |  |  |  |  |
| Individuals with cancer / cancer treatment–related pain and a previous history of opioid misuse / abuse / addiction |  | | | | | 0% | | 7% | 16% | 54% | 22% | 0% | | 6% | 9% | 75% | 10% | 0.763 | <0.001 |
|  |  |  |  |  |  | 7% | | | 16% | 76% | | 6% | | | 9% | 85% | |  |  |
| Individuals with cancer / cancer treatment–related pain and ongoing problems of opioid misuse / abuse / addiction |  |  |  |  |  | 1% | | 11% | 21% | 46% | 21% | 0% | | 9% | 14% | 70% | 8% | 0.753 | <0.001 |
|  |  |  |  |  |  | 12% | | | 21% | 67% | | 9% | | | 14% | 78% | |  |  |
| **SPCS services should have more education and / or appropriate training in the following areas:** | | | | | | | | | | | | | | | | | | | |
| Interventional pain techniques |  | | | | | 5% | | 5% | 7% | 35% | 48% | 3% | | 3% | 4% | 38% | 54% | 0.802 | <0.001 |
|  |  |  |  |  |  | 10% | | | 8% | 83% | | 6% | | | 4% | 92% | |  |  |
| Physiotherapy interventions |  |  |  |  |  | 2% | | 1% | 14% | 43% | 40% | 1% | | 1% | 5% | 58% | 35% | 0.780 | <0.001 |
|  |  |  |  |  |  | 3% | | | 14% | 83% | | 2% | | | 5% | 93% | |  |  |
| Occupational therapy interventions |  |  |  |  |  | 2% | | 5% | 19% | 40% | 35% | 1% | | 6% | 10% | 51% | 31% | 0.830 | <0.001 |
|  |  |  |  |  |  | 7% | | | 19% | 75% | | 7% | | | 10% | 82% | |  |  |
| Transcutaneous electrical nerve stimulation (TENS), scrambler therapy |  |  |  |  |  | 4% | | 5% | 15% | 47% | 30% | 3% | | 4% | 14% | 56% | 24% | 0.774 | <0.001 |
|  |  |  |  |  |  | 9% | | | 15% | 77% | | 7% | | | 14% | 80% | |  |  |
| Acupuncture, acupressure |  |  |  |  |  | 5% | | 9% | 28% | 32% | 26% | 4% | | 10% | 26% | 36% | 24% | 0.835 | <0.001 |
|  |  |  |  |  |  | 14% | | | 29% | 58% | | 14% | | | 26% | 60% | |  |  |
| Psychological and behavioural therapies / interventions |  |  |  |  |  | 1% | | 0% | 11% | 44% | 43% | 0% | | 0% | 9% | 49% | 43% | 0.857 | <0.001 |
|  |  |  |  |  |  | 1% | | | 11% | 87% | | 0% | | | 9% | 92% | |  |  |
| Complementary and alternative therapies |  |  |  |  |  | 9% | | 19% | 25% | 22% | 26% | 6% | | 19% | 20% | 19% | 36% | 0.835 | <0.001 |
|  |  |  |  |  |  | 28% | | | 25% | 48% | | 25% | | | 20% | 55% | |  |  |
| Pharmacological interventions in addiction recovery |  |  |  |  |  | 5% | | 6% | 12% | 46% | 31% | 1% | | 5% | 10% | 60% | 24% | 0.742 | <0.001 |
|  |  |  |  |  |  | 11% | | | 11% | 77% | | 6% | | | 10% | 84% | |  |  |
| Psychosocial and behavioural interventions in addiction recovery |  |  |  |  |  | 7% | | 10% | 19% | 36% | 28% | 4% | | 8% | 20% | 48% | 21% | 0.782 | <0.001 |
|  |  |  |  |  |  | 17% | | | 18% | 66% | | 12% | | | 20% | 69% | |  |  |
| **SPCS should manage the following types of non-pain symptoms / physical problems:** | | | | | | | | | | | | | | | | | | | |
| ﻿Acute cancer-related symptoms / physical problems (lasting < 3 months) | 0% | 4% | 8% | 20% | 68% | 1% | | 1% | 4% | 16% | 78% |  | | | | | | 0.678** | <0.001 |
|  | 4% | | 8% | 88% | | 2% | | | 4% | 94% | |  |  |  |  |  |  |  |  |
| Acute cancer treatment-related symptoms / physical problems (lasting < 3 months) | 2% | 4% | 17% | 21% | 56% | 4% | | 1% | 9% | 20% | 67% |  |  |  |  |  |  | 0.686** | <0.001 |
|  | 6% | | 17% | 77% | | 5% | | | 9% | 87% | |  |  |  |  |  |  |  |  |
| Acute symptoms / physical problems related to non-cancer causes (lasting < 3 months) | 6% | 8% | 20% | 27% | 38% | 5% | | 7% | 11% | 31% | 46% |  |  |  |  |  |  | 0.659** | <0.001 |
|  | 14% | | 20% | 66% | | 13% | | | 11% | 77% | |  |  |  |  |  |  |  |  |
| Chronic cancer-related symptoms / physical problems (lasting ≥ 3 months) | 0% | 2% | 10% | 13% | 75% | 1% | | 0% | 4% | 15% | 80% |  |  |  |  |  |  | 0.758** | <0.001 |
|  | 2% | | 10% | 88% | | 1% | | | 4% | 95% | |  |  |  |  |  |  |  |  |
| Chronic cancer treatment-related symptoms / physical problems (lasting ≥ 3 months) | 1% | 2% | 15% | 24% | 57% | 1% | | 1% | 10% | 25% | 63% |  |  |  |  |  |  | 0.702** | <0.001 |
|  | 3% | | 15% | 81% | | 2% | | | 10% | 88% | |  |  |  |  |  |  |  |  |
| Chronic symptoms / physical problems related to non-cancer causes (lasting ≥ 3 months) | 7% | 12% | 21% | 20% | 39% | 6% | | 14% | 16% | 25% | 40% |  |  |  |  |  |  | 0.860** | <0.001 |
|  | 19% | | 21% | 59% | | 20% | | | 16% | 65% | |  |  |  |  |  |  |  |  |
| **SPCS generally have the knowledge and skills to manage the following:** | | | | | | | | | | | | | | | | | | | |
| ﻿Acute cancer-related symptoms / physical problems (lasting < 3 months) | 0% | 1% | 1% | 31% | 67% | 0% | | 0% | 2% | 25% | 73% |  | | | | | | 0.687** | <0.001 |
|  | 1% | | 1% | 98% | | 0% | | | 2% | 98% | |  |  |  |  |  |  |  |  |
| Acute cancer treatment-related symptoms / physical problems (lasting < 3 months) | 0% | 5% | 7% | 40% | 48% | 0% | | 1% | 5% | 38% | 56% |  |  |  |  |  |  | 0.711** | <0.001 |
|  | 5% | | 7% | 88% | | 1% | | | 5% | 94% | |  |  |  |  |  |  |  |  |
| Acute symptoms / physical problems related to non-cancer causes (lasting < 3 months) | 2% | 7% | 18% | 35% | 38% | 1% | | 5% | 16% | 36% | 42% |  |  |  |  |  |  | 0.735** | <0.001 |
|  | 9% | | 18% | 73% | | 6% | | | 16% | 78% | |  |  |  |  |  |  |  |  |
| Chronic cancer-related symptoms / physical problems (lasting ≥ 3 months) | 0% | 1% | 4% | 27% | 68% | 0% | | 2% | 2% | 22% | 73% |  |  |  |  |  |  | 0.756** | <0.001 |
|  | 1% | | 4% | 95% | | 2% | | | 2% | 95% | |  |  |  |  |  |  |  |  |
| Chronic cancer treatment-related symptoms / physical problems (lasting ≥ 3 months) | 0% | 8% | 6% | 36% | 50% | 0% | | 6% | 5% | 30% | 59% |  |  |  |  |  |  | 0.794** | <0.001 |
|  | 8% | | 6% | 86% | | 6% | | | 5% | 89% | |  |  |  |  |  |  |  |  |
| Chronic symptoms / physical problems related to non-cancer causes (lasting ≥ 3 months) | 8% | 14% | 8% | 36% | 33% | 6% | | 12% | 9% | 43% | 30% |  |  |  |  |  |  | 0.818** | <0.001 |
|  | 22% | | 8% | 69% | | 18% | | | 9% | 73% | |  |  |  |  |  |  |  |  |
| **SPCS services should have more training in the following areas:** | | | | | | | | | | | | | | | | | | | |
| Assessment of acute side effects of anticancer treatment (especially novel anticancer treatments) |  | | | | | 0% | | 4% | 1% | 26% | 69% | 0% | | 1% | 0% | 11% | 88% | 0.570 | <0.001 |
|  |  |  |  |  |  | 4% | | | 1% | 95% | | 1% | | | 0% | 99% | |  |  |
| Management of acute side effects of anticancer treatment (especially novel anticancer treatments) |  |  |  |  |  | 2% | | 2% | 2% | 30% | 63% | 0% | | 4% | 0% | 15% | 81% | 0.646 | <0.001 |
|  |  |  |  |  |  | 4% | | | 2% | 93% | | 4% | | | 0% | 96% | |  |  |
| Assessment of chronic / long-term effects of anticancer treatment (especially novel anticancer treatments) |  |  |  |  |  | 0% | | 2% | 4% | 2% | 73% | 0% | | 1% | 0% | 15% | 84% | 0.754 | <0.001 |
|  |  |  |  |  |  | 2% | | | 4% | 95% | | 1% | | | 0% | 99% | |  |  |
| Management of chronic / long-term effects of anticancer treatment (especially novel anticancer treatments) |  |  |  |  |  | 0% | | 2% | 4% | 30% | 64% | 0% | | 1% | 3% | 19% | 78% | 0.751 | <0.001 |
|  |  |  |  |  |  | 2% | | | 4% | 94% | | 1% | | | 3% | 97% | |  |  |
| Assessment of late / delayed effects of anticancer treatment (especially novel anticancer treatments) |  |  |  |  |  | 0% | | 4% | 2% | 31% | 63% | 0% | | 1% | 0% | 23% | 76% | 0.770 | <0.001 |
|  |  |  |  |  |  | 4% | | | 2% | 94% | | 1% | | | 0% | 99% | |  |  |
| Management of late / delayed effects of anticancer treatment (especially novel anticancer treatments) |  |  |  |  |  | 0% | | 4% | 2% | 36% | 58% | 0% | | 3% | 1% | 25% | 71% | 0.789 | <0.001 |
|  |  |  |  |  |  | 4% | | | 2% | 94% | | 3% | | | 1% | 96% | |  |  |

| **Key** |  |  |  |  |
| --- | --- | --- | --- | --- |
| SD | Strongly disagree | | rho | Spearman’s rank correlation coefficient between relevant subsequent rounds |
| MD | Moderately disagree | | * | Correlation is significant at the 0.05 level (2-tailed) |
| N | Neither agree nor disagree | | ** | Correlation is significant at the 0.01 level (2-tailed) |
| MA | Moderately agree | | Shaded box | Consensus reached (≥75%) |
| SA | Strongly agree | |  |  |
| SPC | Specialist palliative care | |  |  |
| SPCS | Specialist palliative care services | | |  |
|  |  |  |  |  |

S1b. Extended role of Specialist Palliative Care for individuals with a history of cancer who have completed anticancer treatment and have no evidence of disease – SPC group results

| **Statement** | **Round 1** | | | | | **Round 2** | | | | | | **Round 3** | | | | | **rho** | **p value** |
| --- | --- | --- | --- | --- | --- | --- | --- | --- | --- | --- | --- | --- | --- | --- | --- | --- | --- | --- |
|  | **SD** | **MD** | **N** | **MA** | **SA** | **SD** | | **MD** | **N** | **MA** | **SA** | **SD** | **MD** | **N** | **MA** | **SA** |  |  |
| **SPCS should have a role in supporting the following components of care** | | | | | | | | | | | | | | | | | | |
| ﻿Prevention of recurrent and new cancers | 42% | 36% | 13% | 5% | 5% |  | | | | | | | | | | | | |
|  | 78% | | 13% | 10% | |  |  |  |  |  |  |  |  |  |  |  |  |  |
| ﻿Prevention of other late effects | 25% | 24% | 12% | 20% | 19% |  |  |  |  |  |  |  |  |  |  |  |  |  |
|  | 49% | | 12% | 39% | |  |  |  |  |  |  |  |  |  |  |  |  |  |
| ﻿Surveillance for cancer spread, recurrence, or second cancers | 46% | 23% | 15% | 11% | 5% |  |  |  |  |  |  |  |  |  |  |  |  |  |
|  | 69% | | 15% | 16% | |  |  |  |  |  |  |  |  |  |  |  |  |  |
| ﻿Surveillance of physical late effects | 25% | 19% | 13% | 23% | 20% |  |  |  |  |  |  |  |  |  |  |  |  |  |
|  | 44% | | 13% | 43% | |  |  |  |  |  |  |  |  |  |  |  |  |  |
| ﻿Surveillance of psychosocial late effects | 25% | 17% | 15% | 23% | 20% |  |  |  |  |  |  |  |  |  |  |  |  |  |
|  | 42% | | 15% | 43% | |  |  |  |  |  |  |  |  |  |  |  |  |  |
| ﻿Intervention for pain as a late effect | 12% | 11% | 7% | 35% | 36% |  |  |  |  |  |  |  |  |  |  |  |  |  |
|  | 23% | | 7% | 71% | |  |  |  |  |  |  |  |  |  |  |  |  |  |
| ﻿Intervention for other physical late effects | 12% | 12% | 10% | 37% | 30% |  |  |  |  |  |  |  |  |  |  |  |  |  |
|  | 24% | | 10% | 67% | |  |  |  |  |  |  |  |  |  |  |  |  |  |
| ﻿Intervention for psychosocial late effects | 13% | 8% | 12% | 38% | 29% |  |  |  |  |  |  |  |  |  |  |  |  |  |
|  | 21% | | 12% | 67% | |  |  |  |  |  |  |  |  |  |  |  |  |  |
| ﻿Managing “fear of cancer recurrence” | 25% | 15% | 18% | 27% | 14% |  |  |  |  |  |  |  |  |  |  |  |  |  |
|  | 40% | | 18% | 41% | |  |  |  |  |  |  |  |  |  |  |  |  |  |
| ﻿Rehabilitation | 26% | 29% | 10% | 25% | 11% |  |  |  |  |  |  |  |  |  |  |  |  |  |
|  | 55% | | 10% | 36% | |  |  |  |  |  |  |  |  |  |  |  |  |  |
| ﻿Supporting occupational issues / return to work | 27% | 31% | 17% | 18% | 7% |  |  |  |  |  |  |  |  |  |  |  |  |  |
|  | 58% | | 17% | 25% | |  |  |  |  |  |  |  |  |  |  |  |  |  |
| ﻿Managing financial problems (“financial toxicity”) | 33% | 29% | 23% | 11% | 5% |  |  |  |  |  |  |  |  |  |  |  |  |  |
|  | 62% | | 23% | 16% | |  |  |  |  |  |  |  |  |  |  |  |  |  |
| ﻿Supporting concerns related to insurance | 33% | 31% | 20% | 8% | 7% |  |  |  |  |  |  |  |  |  |  |  |  |  |
|  | 64% | | 20% | 15% | |  |  |  |  |  |  |  |  |  |  |  |  |  |
| ﻿Supporting caregivers | 23% | 21% | 14% | 27% | 14% |  |  |  |  |  |  |  |  |  |  |  |  |  |
|  | 44% | | 14% | 41% | |  |  |  |  |  |  |  |  |  |  |  |  |  |
| ﻿Supporting coordination between specialists and primary care providers | 26% | 21% | 13% | 20% | 19% |  |  |  |  |  |  |  |  |  |  |  |  |  |
|  | 47% | | 13% | 39% | |  |  |  |  |  |  |  |  |  |  |  |  |  |
| Prevention of second cancers |  | | | | | 58% | | 27% | 9% | 2% | 4% | 73% | 16% | 8% | 0% | 4% | 0.763 | <0.001 |
|  |  |  |  |  |  | 85% | | | 9% | 6% | | 89% | | 8% | 4% | |  |  |
| ﻿Surveillance for recurrence or second cancers |  |  |  |  |  | 62% | | 21% | 9% | 5% | 4% | 79% | 9% | 5% | 4% | 4% | 0.730 | <0.001 |
|  |  |  |  |  |  | 83% | | | 9% | 9% | | 88% | | 5% | 8% | |  |  |
| ﻿Surveillance of physical "late effects" |  |  |  |  |  | 38% | | 20% | 4% | 22% | 16% | 55% | 11% | 3% | 19% | 13% | 0.810 | <0.001 |
|  |  |  |  |  |  | 58% | | | 4% | 38% | | 66% | | 3% | 32% | |  |  |
| ﻿Surveillance of psychological distress |  |  |  |  |  | 37% | | 16% | 7% | 22% | 17% | 54% | 11% | 4% | 19% | 13% | 0.804 | <0.001 |
|  |  |  |  |  |  | 53% | | | 8% | 39% | | 66% | | 4% | 32% | |  |  |
| Management of pain |  |  |  |  |  | 14% | | 6% | 7% | 35% | 38% | 15% | 5% | 5% | 33% | 43% | 0.943 | <0.001 |
|  |  |  |  |  |  | 20% | | | 8% | 73% | | 20% | | 5% | 76% | |  |  |
| Management of other physical symptoms / problems |  |  |  |  |  | 14% | | 7% | 9% | 44% | 26% | 16% | 5% | 6% | 48% | 25% | 0.927 | <0.001 |
|  |  |  |  |  |  | 21% | | | 9% | 70% | | 21% | | 6% | 73% | |  |  |
| Management of psychological distress |  |  |  |  |  | 15% | | 5% | 11% | 44% | 25% | 14% | 4% | 11% | 48% | 24% | 0.956 | <0.001 |
|  |  |  |  |  |  | 20% | | | 11% | 69% | | 18% | | 11% | 72% | |  |  |
| ﻿Managing “fear of cancer recurrence” |  |  |  |  |  | 30% | | 14% | 12% | 32% | 12% | 38% | 5% | 9% | 38% | 11% | 0.908 | <0.001 |
|  |  |  |  |  |  | 44% | | | 13% | 44% | | 43% | | 9% | 49% | |  |  |
| Rehabilitation |  |  |  |  |  | 32% | | 27% | 7% | 23% | 10% | 46% | 20% | 5% | 19% | 10% | 0.829 | <0.001 |
|  |  |  |  |  |  | 59% | | | 8% | 33% | | 66% | | 5% | 29% | |  |  |
| ﻿Supporting occupational issues / return to work |  |  |  |  |  | 36% | | 30% | 16% | 12% | 6% | 49% | 21% | 15% | 9% | 6% | 0.847 | <0.001 |
|  |  |  |  |  |  | 66% | | | 16% | 18% | | 70% | | 15% | 15% | |  |  |
| Managing financial problems (“financial toxicity”) |  |  |  |  |  | 46% | | 27% | 15% | 7% | 5% | 60% | 19% | 10% | 6% | 5% | 0.817 | <0.001 |
|  |  |  |  |  |  | 73% | | | 15% | 12% | | 79% | | 10% | 11% | |  |  |
| ﻿Supporting caregivers |  |  |  |  |  | 30% | | 16% | 11% | 31% | 12% | 40% | 11% | 9% | 28% | 13% | 0.835 | <0.001 |
|  |  |  |  |  |  | 46% | | | 11% | 33% | | 51% | | 9% | 41% | |  |  |
| ﻿Coordination between specialists and primary care providers |  |  |  |  |  | 40% | | 16% | 9% | 19% | 17% | 55% | 8% | 9% | 15% | 14% | 0.849 | <0.001 |
|  |  |  |  |  |  | 56% | | | 9% | 36% | | 63% | | 9% | 29% | |  |  |
| Developing "survivorship care plans" |  |  |  |  |  | 33% | | 12% | 20% | 25% | 10% | 48% | 9% | 14% | 23% | 8% | 0.765 | <0.001 |
|  |  |  |  |  |  | 45% | | | 20% | 35% | | 57% | | 14% | 31% | |  |  |
| **SPCS generally have the knowledge and skills to support the following components of care:** | | | | | | | | | | | | | | | | | | |
| ﻿Prevention of recurrent and new cancers | 40% | 30% | 13% | 13% | 4% |  | | | | | | | | | | | | |
|  | 70% | | 13% | 17% | |  |  |  |  |  |  |  |  |  |  |  |  |  |
| ﻿Prevention of other late effects | 30% | 24% | 12% | 19% | 15% |  |  |  |  |  |  |  |  |  |  |  |  |  |
|  | 54% | | 12% | 34% | |  |  |  |  |  |  |  |  |  |  |  |  |  |
| ﻿Surveillance for cancer spread, recurrence, or second cancers | 40% | 31% | 8% | 14% | 6% |  |  |  |  |  |  |  |  |  |  |  |  |  |
|  | 71% | | 8% | 29% | |  |  |  |  |  |  |  |  |  |  |  |  |  |
| ﻿Surveillance of physical late effects | 21% | 18% | 8% | 29% | 24% |  |  |  |  |  |  |  |  |  |  |  |  |  |
|  | 39% | | 8% | 53% | |  |  |  |  |  |  |  |  |  |  |  |  |  |
| ﻿Surveillance of psychosocial late effects | 20% | 14% | 8% | 32% | 25% |  |  |  |  |  |  |  |  |  |  |  |  |  |
|  | 34% | | 8% | 57% | |  |  |  |  |  |  |  |  |  |  |  |  |  |
| ﻿Intervention for pain as a late effect | 8% | 6% | 11% | 32% | 43% |  |  |  |  |  |  |  |  |  |  |  |  |  |
|  | 14% | | 11% | 75% | |  |  |  |  |  |  |  |  |  |  |  |  |  |
| ﻿Intervention for other physical late effects | 8% | 8% | 12% | 37% | 35% |  |  |  |  |  |  |  |  |  |  |  |  |  |
|  | 16% | | 12% | 72% | |  |  |  |  |  |  |  |  |  |  |  |  |  |
| ﻿Intervention for psychosocial late effects | 11% | 11% | 8% | 38% | 32% |  |  |  |  |  |  |  |  |  |  |  |  |  |
|  | 22% | | 8% | 70% | |  |  |  |  |  |  |  |  |  |  |  |  |  |
| ﻿Managing “fear of cancer recurrence” | 13% | 19% | 24% | 25% | 19% |  |  |  |  |  |  |  |  |  |  |  |  |  |
|  | 32% | | 24% | 44% | |  |  |  |  |  |  |  |  |  |  |  |  |  |
| ﻿Rehabilitation | 20% | 21% | 20% | 30% | 8% |  |  |  |  |  |  |  |  |  |  |  |  |  |
|  | 41% | | 20% | 38% | |  |  |  |  |  |  |  |  |  |  |  |  |  |
| ﻿Supporting occupational issues / return to work | 26% | 27% | 15% | 24% | 7% |  |  |  |  |  |  |  |  |  |  |  |  |  |
|  | 53% | | 15% | 31% | |  |  |  |  |  |  |  |  |  |  |  |  |  |
| ﻿Managing financial problems (“financial toxicity”) | 29% | 27% | 17% | 20% | 7% |  |  |  |  |  |  |  |  |  |  |  |  |  |
|  | 56% | | 17% | 27% | |  |  |  |  |  |  |  |  |  |  |  |  |  |
| ﻿Supporting concerns related to insurance | 32% | 30% | 17% | 17% | 5% |  |  |  |  |  |  |  |  |  |  |  |  |  |
|  | 62% | | 17% | 23% | |  |  |  |  |  |  |  |  |  |  |  |  |  |
| ﻿Supporting caregivers | 12% | 7% | 13% | 40% | 27% |  |  |  |  |  |  |  |  |  |  |  |  |  |
|  | 19% | | 13% | 67% | |  |  |  |  |  |  |  |  |  |  |  |  |  |
| ﻿Supporting coordination between specialists and primary care providers | 12% | 12% | 15% | 33% | 27% |  |  |  |  |  |  |  |  |  |  |  |  |  |
|  | 24% | | 15% | 60% | |  |  |  |  |  |  |  |  |  |  |  |  |  |
| Prevention of second cancers |  | | | | | 57% | | 23% | 9% | 7% | 4% | 70% | 20% | 4% | 5% | 1% | 0.692 | <0.001 |
|  |  |  |  |  |  | 80% | | | 9% | 11% | | 90% | | 4% | 6% | |  |  |
| ﻿Surveillance for recurrence or second cancers |  |  |  |  |  | 53% | | 25% | 5% | 11% | 6% | 68% | 18% | 4% | 8% | 4% | 0.734 | <0.001 |
|  |  |  |  |  |  | 78% | | | 5% | 17% | | 86% | | 4% | 12% | |  |  |
| ﻿Surveillance of physical "late effects" |  |  |  |  |  | 25% | | 15% | 9% | 31% | 21% | 28% | 11% | 4% | 40% | 18% | 0.920 | <0.001 |
|  |  |  |  |  |  | 40% | | | 9% | 52% | | 39% | | 4% | 58% | |  |  |
| ﻿Surveillance of psychological distress |  |  |  |  |  | 22% | | 9% | 10% | 36% | 23% | 23% | 8% | 6% | 46% | 18% | 0.908 | <0.001 |
|  |  |  |  |  |  | 31% | | | 10% | 59% | | 31% | | 6% | 64% | |  |  |
| Management of pain |  |  |  |  |  | 6% | | 4% | 7% | 35% | 48% | 4% | 1% | 8% | 35% | 53% | 0.862 | <0.001 |
|  |  |  |  |  |  | 10% | | | 7% | 83% | | 5% | | 8% | 88% | |  |  |
| Management of other physical symptoms / problems |  |  |  |  |  | 6% | | 4% | 10% | 43% | 37% | 4% | 0% | 10% | 51% | 35% | 0.846 | <0.001 |
|  |  |  |  |  |  | 10% | | | 10% | 80% | | 4% | | 10% | 86% | |  |  |
| Management of psychological distress |  |  |  |  |  | 7% | | 9% | 7% | 44% | 32% | 6% | 5% | 6% | 58% | 25% | 0.818 | <0.001 |
|  |  |  |  |  |  | 16% | | | 7% | 76% | | 11% | | 6% | 83% | |  |  |
| ﻿Managing “fear of cancer recurrence” |  |  |  |  |  | 11% | | 20% | 25% | 28% | 16% | 9% | 18% | 20% | 39% | 15% | 0.886 | <0.001 |
|  |  |  |  |  |  | 31% | | | 25% | 44% | | 27% | | 20% | 54% | |  |  |
| Rehabilitation |  |  |  |  |  | 17% | | 23% | 22% | 31% | 6% | 16% | 18% | 24% | 39% | 4% | 0.871 | <0.001 |
|  |  |  |  |  |  | 40% | | | 22% | 37% | | 34% | | 24% | 43% | |  |  |
| ﻿Supporting occupational issues / return to work |  |  |  |  |  | 27% | | 35% | 11% | 20% | 7% | 26% | 41% | 8% | 18% | 8% | 0.929 | <0.001 |
|  |  |  |  |  |  | 62% | | | 11% | 27% | | 67% | | 8% | 26% | |  |  |
| Managing financial problems (“financial toxicity”) |  |  |  |  |  | 32% | | 37% | 10% | 17% | 4% | 33% | 43% | 8% | 14% | 4% | 0.853 | <0.001 |
|  |  |  |  |  |  | 69% | | | 10% | 21% | | 76% | | 8% | 18% | |  |  |
| ﻿Supporting caregivers |  |  |  |  |  | 12% | | 2% | 12% | 49% | 23% | 14% | 1% | 8% | 59% | 19% | 0.912 | <0.001 |
|  |  |  |  |  |  | 14% | | | 12% | 72% | | 15% | | 8% | 78% | |  |  |
| ﻿Coordination between specialists and primary care providers |  |  |  |  |  | 11% | | 10% | 12% | 42% | 25% | 11% | 6% | 11% | 54% | 18% | 0.909 | <0.001 |
|  |  |  |  |  |  | 21% | | | 12% | 67% | | 17% | | 11% | 72% | |  |  |
| Developing "survivorship care plans" |  |  |  |  |  | 32% | | 19% | 19% | 25% | 6% | 41% | 11% | 18% | 26% | 4% | 0.797 | <0.001 |
|  |  |  |  |  |  | 51% | | | 19% | 31% | | 52% | | 18% | 30% | |  |  |
| **Barriers to extending the input of SPCS to this group:** | | | | | | | | | | | | | | | | | | |
| Financial resources | 4% | 11% | 11% | 29% | 46% | 0% | | 9% | 10% | 25% | 57% |  | | | | | 0.812** | <0.001 |
|  | 15% | | 11% | 75% | | 9% | | | 10% | 82% | |  |  |  |  |  |  |  |
| Human resources | 5% | 5% | 6% | 24% | 61% | 0% | | 2% | 7% | 21% | 69% |  |  |  |  |  | 0.761** | <0.001 |
|  | 10% | | 6% | 85% | | 2% | | | 7% | 90% | |  |  |  |  |  |  |  |
| Other resources | 6% | 2% | 29% | 25% | 38% |  | |  |  |  |  |  |  |  |  |  |  |  |
|  | 8% | | 29% | 63% | |  | |  |  |  |  |  |  |  |  |  |  |  |
| Lack of relevant education and training (e.g. management of chronic cancer treatment-related symptoms / problems) | 8% | 10% | 8% | 37% | 37% | 4% | | 7% | 9% | 40% | 41% |  |  |  |  |  | 0.795** | <0.001 |
|  | 18% | | 8% | 74% | | 11% | | | 9% | 81% | |  |  |  |  |  |  |  |
| Lack of willingness from palliative care professionals | 17% | 11% | 23% | 27% | 23% | 14% | | 7% | 23% | 35% | 21% |  |  |  |  |  | 0.938** | <0.001 |
|  | 18% | | 23% | 50% | | 21% | | | 23% | 56% | |  |  |  |  |  |  |  |
| Lack of willingness from oncology professionals | 7% | 11% | 27% | 36% | 19% | 5% | | 9% | 26% | 48% | 12% |  |  |  |  |  | 0.827** | <0.001 |
|  | 18% | | 27% | 55% | | 14% | | | 26% | 60% | |  |  |  |  |  |  |  |
| Lack of willingness from general practitioners | 10% | 11% | 36% | 31% | 13% | 7% | | 10% | 41% | 32% | 10% |  |  |  |  |  | 0.900** | <0.001 |
|  | 21% | | 36% | 44% | | 17% | | | 41% | 42% | |  |  |  |  |  |  |  |
| Lack of willingness from patients and families | 12% | 27% | 21% | 27% | 12% | 9% | | 22% | 25% | 33% | 11% |  |  |  |  |  | 0.938** | <0.001 |
|  | 39% | | 21% | 39% | | 31% | | | 25% | 44% | |  |  |  |  |  |  |  |
| Misperceptions about the nature of palliative care | 4% | 4% | 19% | 24% | 50% | 2% | | 2% | 20% | 19% | 57% |  |  |  |  |  | 0.903** | <0.001 |
|  | 8% | | 19% | 74% | | 4% | | | 20% | 76% | |  |  |  |  |  |  |  |
| Time resources |  | | | | | 0% | 4% | | 15% | 22% | 59% | 0% | 5% | 10% | 16% | 69% | 0.823 | <0.001 |
|  |  |  |  |  |  | 4% | | | 15% | 81% | | 5% | | 10% | 85% | |  |  |
| **Type of service** | | | | | | | | | | | | | | | | | | |
| A dedicated multidisciplinary specialist team is required |  | | | | | 6% | | 6% | 9% | 37% | 42% | 1% | 5% | 5% | 30% | 59% | 0.754 | <0.001 |
|  |  |  |  |  |  | 12% | | | 9% | 79% | | 6% | | 5% | 89% | |  |  |
| Specialist palliative care services should lead this team |  |  |  |  |  | 44% | | 17% | 19% | 14% | 6% | 63% | 13% | 14% | 8% | 4% | 0.752 | <0.001 |
|  |  |  |  |  |  | 61% | | | 19% | 20% | | 76% | | 14% | 12% | |  |  |
| Specialist palliative care services should be a core member of this team i.e. routinely involved in care |  |  |  |  |  | 22% | | 22% | 15% | 21% | 20% | 26% | 26% | 9% | 21% | 18% | 0.923 | <0.001 |
|  |  |  |  |  |  | 44% | | | 15% | 41% | | 52% | | 9% | 39% | |  |  |
| Specialist palliative care services should be an extended member of this team i.e. support with relevant problems / issues |  |  |  |  |  | 11% | | 11% | 11% | 38% | 28% | 9% | 10% | 8% | 54% | 20% | 0.787 | <0.001 |
|  |  |  |  |  |  | 22% | | | 11% | 66% | | 19% | | 8% | 74% | |  |  |
| Specialist palliative care services should not contribute to this team |  |  |  |  |  | 53% | | 20% | 14% | 6% | 7% | 66% | 14% | 10% | 3% | 8% | 0.77 | <0.001 |
|  |  |  |  |  |  | 73% | | | 14% | 13% | | 80% | | 10% | 11% | |  |  |

| **Key** |  |  |  |  |
| --- | --- | --- | --- | --- |
| SD | Strongly disagree | | rho | Spearman’s rank correlation coefficient between relevant subsequent rounds |
| MD | Moderately disagree | | * | Correlation is significant at the 0.05 level (2-tailed) |
| N | Neither agree nor disagree | | ** | Correlation is significant at the 0.01 level (2-tailed) |
| MA | Moderately agree | | Shaded box | Consensus reached (≥75%) |
| SA | Strongly agree | |  |  |
| SPC | Specialist palliative care | |  |  |
| SPCS | Specialist palliative care services | | |  |
|  |  |  |  |  |

# Supplementary Tables S2a-b: Oncology (Onc) expert group results

S2a Definition and core functions of Specialist Palliative Care (SPC) – ONC group results

| **Statement** | **Round 1** | | | | | **Round 2** | | | | | | **Round 3** | | | | | | **rho** | **p value** |
| --- | --- | --- | --- | --- | --- | --- | --- | --- | --- | --- | --- | --- | --- | --- | --- | --- | --- | --- | --- |
|  | **SD** | **MD** | **N** | **MA** | **SA** | **SD** | | **MD** | **N** | **MA** | **SA** | **SD** | | **MD** | **N** | **MA** | **SA** |  |  |
| **Section 1: Definition and core functions of Specialist Palliative Care (SPC)** | | | | | | | | | | | | | | | | | | | |
| **Core functions of SPCS** | | | | | | | | | | | | | | | | | | | |
| ﻿Management of pain | 0% | 0% | 4% | 17% | 79% | 0% | | 0% | 2% | 11% | 87% |  | | | | | | 0.754^**^ | <0.001 |
|  | 0% | | 4% | 96% | | 0% | | | 2% | 98% | |  |  |  |  |  |  |  |  |
| Management of other physical symptoms | 0% | 0% | 6% | 19% | 75% | 0% | | 0% | 4% | 9% | 87% |  |  |  |  |  |  | 0.696** | <0.001 |
|  | 0% | | 6% | 94% | | 0% | | | 4% | 96% | |  |  |  |  |  |  |  |  |
| ﻿﻿Management of psychological problems | 0% | 0% | 6% | 38% | 57% | 0% | | 0% | 4% | 38% | 58% |  |  |  |  |  |  | 0.893** | <0.001 |
|  | 0% | | 6% | 95% | | 0% | | | 4% | 96% | |  |  |  |  |  |  |  |  |
| Emotional support (patient) | 0% | 2% | 4% | 26% | 68% | 0% | | 0% | 2% | 23% | 75% |  |  |  |  |  |  | 0.731** | <0.001 |
|  | 2% | | 4% | 94% | | 0% | | | 2% | 98% | |  |  |  |  |  |  |  |  |
| Emotional support (family) | 0% | 2% | 13% | 26% | 58% | 0% | | 0% | 4% | 32% | 64% |  |  |  |  |  |  | 0.805** | <0.001 |
|  | 2% | | 13% | 84% | | 0% | | | 4% | 96% | |  |  |  |  |  |  |  |  |
| Spiritual support | 0% | 9% | 26% | 34% | 30% | 0% | | 8% | 21% | 47% | 25% |  |  |  |  |  |  | 0.893** | <0.001 |
|  | 8% | | 26% | 64% | | 8% | | | 21% | 72% | |  |  |  |  |  |  |  |  |
| Social assistance | 0% | 8% | 30% | 38% | 25% | 0% | | 8% | 32% | 42% | 19% |  |  |  |  |  |  | 0.837** | <0.001 |
|  | 8% | | 30% | 63% | | 8% | | | 32% | 61% | |  |  |  |  |  |  |  |  |
| Co-ordination of care | 0% | 2% | 11% | 28% | 58% | 0% | | 0% | 4% | 34% | 62% |  |  |  |  |  |  | 0.909** | <0.001 |
|  | 2% | | 11% | 86% | | 0% | | | 4% | 96% | |  |  |  |  |  |  |  |  |
| Advance care planning | 0% | 2% | 8% | 25% | 66% | 0% | | 0% | 2% | 25% | 74% |  |  |  |  |  |  | 0.793** | <0.001 |
|  | 2% | | 8% | 91% | | 0% | | | 2% | 98% | |  |  |  |  |  |  |  |  |
| End-of-life care | 2% | 0% | 2% | 17% | 79% | 0% | | 0% | 0% | 13% | 87% |  |  |  |  |  |  | 0.723** | <0.001 |
|  | 2% | | 2% | 96% | | 0% | | | 0% | 100% | |  |  |  |  |  |  |  |  |
| Bereavement care | 0% | 6% | 21% | 30% | 43% | 0% | | 2% | 17% | 28% | 53% |  |  |  |  |  |  | 0.852** | <0.001 |
|  | 6% | | 21% | 73% | | 2% | | | 17% | 81% | |  |  |  |  |  |  |  |  |
| Rehabilitation | 2% | 11% | 28% | 32% | 26% | 2% | | 9% | 30% | 36% | 23% |  |  |  |  |  |  | 0.951** | <0.001 |
|  | 13% | | 28% | 58% | | 11% | | | 30% | 59% | |  |  |  |  |  |  |  |  |
| Religious support |  | | | | | 6% | | 19% | 43% | 21% | 11% | 6% | | 16% | 57% | 12% | 8% | 0.723** | <0.001 |
|  |  |  |  |  |  | 25% | | | 43% | 32% | | 32% | | | 57% | 20% | |  |  |
| Research |  |  |  |  |  | 0% | | 2% | 19% | 23% | 57% | 0% | | 0% | 6% | 22% | 71% | 0.743** | <0.001 |
|  |  |  |  |  |  | 2% | | | 19% | 80% | | 0% | | | 6% | 93% | |  |  |
| Education (other healthcare professionals) |  |  |  |  |  | 0% | 2% | | 4% | 26% | 68% | 0% | 0% | | 0% | 10% | 90% | 0.559** | <0.001 |
|  |  |  |  |  |  | 2% | | | 4% | 94% | | 0% | | | 0% | 100% | |  |  |
| **SPCS should manage the following types of pain:** | | | | | | | | | | | | | | | | | | | |
| Acute cancer-related pain (pain for < 3 months) | 0% | 4% | 9% | 28% | 58% | 0% | | 2% | 4% | 32% | 62% |  | | | | | | 0.899** | <0.001 |
|  | 4% | | 9% | 86% | | 2% | | | 4% | 94% | |  |  |  |  |  |  |  |  |
| Acute cancer treatment-related pain (pain for < 3 months) | 4% | 4% | 13% | 30% | 49% | 0% | | 4% | 4% | 38% | 55% |  |  |  |  |  |  | 0.853** | <0.001 |
|  | 8% | | 13% | 79% | | 4% | | | 4% | 92% | |  |  |  |  |  |  |  |  |
| Acute pain related to non-cancer causes (pain for < 3 months) | 2% | 15% | 21% | 26% | 36% | 2% | | 11% | 19% | 32% | 36% |  |  |  |  |  |  | 0.884** | <0.001 |
|  | 17% | | 21% | 62% | | 13% | | | 19% | 68% | |  |  |  |  |  |  |  |  |
| Chronic cancer-related pain (pain for≥3 months) | 0% | 0% | 9% | 19% | 72% | 0% | | 0% | 4% | 11% | 85% |  |  |  |  |  |  | 0.678** | <0.001 |
|  | 0% | | 9% | 91% | | 0% | | | 4% | 96% | |  |  |  |  |  |  |  |  |
| Chronic cancer treatment-related pain (pain for≥3 months) | 0% | 0% | 11% | 28% | 60% | 0% | | 0% | 4% | 17% | 79% |  |  |  |  |  |  | 0.619** | <0.001 |
|  | 0% | | 11% | 88% | | 0% | | | 4% | 96% | |  |  |  |  |  |  |  |  |
| Chronic pain related to non-cancer causes (pain for≥3 months) | 4% | 4% | 30% | 19% | 43% | 4% | | 2% | 28% | 17% | 49% |  |  |  |  |  |  | 0.915** | <0.001 |
|  | 8% | | 30% | 62% | | 6% | | | 28% | 66% | |  |  |  |  |  |  |  | |
| Individuals with pain and a previous history of opioid misuse / abuse / addiction | 8% | 8% | 13% | 26% | 45% |  | | | | | |  |  |  |  |  |  |  |  |
|  | 16% | | 13% | 71% | |  |  |  |  |  |  |  |  |  |  |  |  |  |  |
| Individuals with pain and ongoing problems of opioid misuse / abuse / addiction | 8% | 6% | 15% | 30% | 42% |  |  |  |  |  |  |  |  |  |  |  |  |  |  |
|  | 14% | | 15% | 72% | |  |  |  |  |  |  |  |  |  |  |  |  |  |  |
| Chronic cancer / cancer treatment-related pain – in collaboration with the multidisciplinary chronic pain team |  | | | | | 0% | | 2% | 6% | 23% | 70% | 0% | | 2% | 2% | 10% | 86% | 0.636** | <0.001 |
|  |  |  |  |  |  | 2% | | | 6% | 93% | | 2% | | | 2% | 96% | |  |  |
| Individuals with cancer / cancer treatment–related pain and a previous history of opioid misuse / abuse / addiction |  |  |  |  |  | 0% | | 8% | 9% | 45% | 38% | 0% | | 2% | 0% | 67% | 31% | 0.686** | <0.001 |
|  |  |  |  |  |  | 8% | | | 9% | 83% | | 2% | | | 0% | 98% | |  |  |
| Individuals with cancer / cancer treatment–related pain and ongoing problems of opioid misuse / abuse / addiction |  |  |  |  |  | 0% | | 8% | 13% | 40% | 40% | 0% | | 2% | 2% | 49% | 47% | 0.554** | <0.001 |
|  |  |  |  |  |  | 8% | | | 13% | 80% | | 2% | | | 2% | 96% | |  |  |
| Individuals with chronic cancer / cancer treatment–related pain and a previous history of opioid misuse / abuse / addiction – in collaboration with the multidisciplinary chronic pain team |  |  |  |  |  | 0% | | 2% | 9% | 23% | 66% | 0% | | 4% | 6% | 8% | 82% | 0.701** | <0.001 |
|  |  |  |  |  |  | 2% | | | 9% | 89% | | 4% | | | 6% | 90% | |  |  |
| Individuals with chronic cancer / cancer treatment–related pain and ongoing problems of opioid misuse / abuse / addiction – in collaboration with the multidisciplinary chronic pain team |  |  |  |  |  | 0% | | 2% | 6% | 26% | 66% | 0% | | 4% | 2% | 16% | 78% | 0.644** | <0.001 |
|  |  |  |  |  |  | 2% | | | 6% | 92% | | 4% | | | 2% | 94% | |  |  |
| Individuals with cancer / cancer treatment–related pain and a previous history of opioid misuse / abuse / addiction – in collaboration with addiction services |  |  |  |  |  | 0% | | 4% | 11% | 27% | 58% | 0% | | 2% | 4% | 14% | 80% | 0.639** | <0.001 |
|  |  |  |  |  |  | 4% | | | 11% | 75% | | 2% | | | 4% | 94% | |  |  |
| Individuals with cancer / cancer treatment–related pain and ongoing problems of opioid misuse / abuse / addiction – in collaboration with addiction services |  |  |  |  |  | 0% | | 4% | 9% | 25% | 62% | 0% | | 2% | 2% | 16% | 80% | 0.572** | <0.001 |
|  |  |  |  |  |  | 4% | | | 9% | 87% | | 2% | | | 2% | 96% | |  |  |
| **SPCS generally have the knowledge and skills to manage the following:** | | | | | | | | | | | | | | | | | | | |
| Acute cancer-related pain (pain for < 3 months) | 0% | 2% | 8% | 19% | 72% | 0% | | 0% | 6% | 9% | 85% |  | | | | | | 0.714** | <0.001 |
|  | 2% | | 8% | 91% | | 0% | | | 6% | 94% | |  |  |  |  |  |  |  |  |
| Acute cancer treatment-related pain (pain for < 3 months) | 0% | 4% | 8% | 32% | 57% | 0% | | 0% | 6% | 26% | 68% |  |  |  |  |  |  | 0.826** | <0.001 |
|  | 4% | | 8% | 89% | | 0% | | | 6% | 94% | |  |  |  |  |  |  |  |  |
| Acute pain related to non-cancer causes (pain for < 3 months) | 0% | 9% | 11% | 38% | 42% | 0% | | 8% | 9% | 38% | 45% |  |  |  |  |  |  | 0.946** | <0.001 |
|  | 9% | | 11% | 80% | | 8% | | | 9% | 83% | |  |  |  |  |  |  |  |  |
| Chronic cancer-related pain (pain for≥3 months) | 0% | 4% | 6% | 21% | 70% | 0% | | 4% | 2% | 15% | 79% |  |  |  |  |  |  | 0.797** | <0.001 |
|  | 4% | | 6% | 91% | | 4% | | | 2% | 94% | |  |  |  |  |  |  |  |  |
| Chronic cancer treatment-related pain (pain for≥3 months) | 0% | 4% | 8% | 28% | 60% | 0% | | 2% | 2% | 23% | 74% |  |  |  |  |  |  | 0.697** | <0.001 |
|  | 4% | | 8% | 88% | | 2% | | | 2% | 96% | |  |  |  |  |  |  |  |  |
| Chronic pain related to non-cancer causes (pain for≥3 months) | 2% | 9% | 15% | 32% | 42% | 0% | | 9% | 13% | 28% | 49% |  |  |  |  |  |  | 0.900** | <0.001 |
|  | 11% | | 15% | 74% | | 9% | | | 13% | 78% | |  |  |  |  |  |  |  |  |
| Individuals with pain and a previous history of opioid misuse / abuse / addiction | 6% | 13% | 25% | 28% | 28% |  | | | | | |  |  |  |  |  |  |  | |
|  | 19% | | 25% | 56% | |  |  |  |  |  |  |  |  |  |  |  |  |  |  |
| Individuals with pain and ongoing problems of opioid misuse / abuse / addiction | 8% | 11% | 23% | 30% | 28% |  |  |  |  |  |  |  |  |  |  |  |  |  |  |
|  | 19% | | 23% | 58% | |  |  |  |  |  |  |  |  |  |  |  |  |  |  |
| Individuals with cancer / cancer treatment–related pain and a previous history of opioid misuse / abuse / addiction |  | | | | | 0% | | 8% | 13% | 42% | 38% | 0% | | 2% | 4% | 73% | 20% | 0.683** | <0.001 |
|  |  |  |  |  |  | 8% | | | 13% | 80% | | 2% | | | 4% | 93% | |  |  |
| Individuals with cancer / cancer treatment–related pain and ongoing problems of opioid misuse / abuse / addiction |  |  |  |  |  | 2% | | 9% | 13% | 38% | 38% | 0% | | 2% | 4% | 65% | 29% | 0.760** | <0.001 |
|  |  |  |  |  |  | 11% | | | 13% | 76% | | 2% | | | 4% | 94% | |  |  |
| **SPCS services should have more education and / or appropriate training in the following areas:** | | | | | | | | | | | | | | | | | | | |
| Interventional pain techniques |  | | | | | 0% | | 4% | 9% | 34% | 53% | 0% | | 2% | 6% | 24% | 67% | 0.829** | <0.001 |
|  |  |  |  |  |  | 4% | | | 9% | 87% | | 2% | | | 6% | 91% | |  |  |
| Physiotherapy interventions |  |  |  |  |  | 0% | | 9% | 23% | 34% | 34% | 0% | | 6% | 16% | 47% | 31% | 0.890** | <0.001 |
|  |  |  |  |  |  | 9% | | | 23% | 68% | | 6% | | | 16% | 78% | |  |  |
| Occupational therapy interventions |  |  |  |  |  | 0% | | 15% | 21% | 30% | 34% | 0% | | 12% | 14% | 35% | 39% | 0.885** | <0.001 |
|  |  |  |  |  |  | 15% | | | 21% | 64% | | 12% | | | 14% | 74% | |  |  |
| Transcutaneous electrical nerve stimulation (TENS), scrambler therapy |  |  |  |  |  | 0% | | 13% | 21% | 36% | 30% | 0% | | 8% | 18% | 53% | 20% | 0.787** | <0.001 |
|  |  |  |  |  |  | 13% | | | 21% | 66% | | 8% | | | 18% | 73% | |  |  |
| Acupuncture, acupressure |  |  |  |  |  | 4% | | 13% | 21% | 42% | 21% | 2% | | 10% | 22% | 57% | 8% | 0.778** | <0.001 |
|  |  |  |  |  |  | 17% | | | 21% | 63% | | 12% | | | 22% | 65% | |  |  |
| Psychological and behavioural therapies / interventions |  |  |  |  |  | 2% | | 8% | 8% | 51% | 32% | 2% | | 6% | 2% | 76% | 14% | 0.719** | <0.001 |
|  |  |  |  |  |  | 10% | | | 8% | 83% | | 8% | | | 2% | 90% | |  |  |
| Complementary and alternative therapies |  |  |  |  |  | 9% | | 9% | 28% | 34% | 19% | 4% | | 8% | 29% | 51% | 8% | 0.761** | <0.001 |
|  |  |  |  |  |  | 18% | | | 28% | 53% | | 12% | | | 29% | 59% | |  |  |
| Pharmacological interventions in addiction recovery |  |  |  |  |  | 0% | | 9% | 21% | 40% | 30% | 0% | | 6% | 18% | 67% | 8% | 0.613** | <0.001 |
|  |  |  |  |  |  | 9% | | | 21% | 70% | | 6% | | | 18% | 75% | |  |  |
| Psychosocial and behavioural interventions in addiction recovery |  |  |  |  |  | 2% | | 13% | 23% | 38% | 25% | 2% | | 10% | 20% | 63% | 4% | 0.724** | <0.001 |
|  |  |  |  |  |  | 15% | | | 23% | 63% | | 12% | | | 20% | 67% | |  |  |
| **SPCS should manage the following types of non-pain symptoms / physical problems:** | | | | | | | | | | | | | | | | | | | |
| ﻿Acute cancer-related symptoms / physical problems (lasting < 3 months) | 0% | 4% | 11% | 28% | 57% | 0% | | 0% | 13% | 23% | 64% |  | | | | | | 0.648** | <0.001 |
|  | 4% | | 11% | 85% | | 0% | | | 13% | 87% | |  |  |  |  |  |  |  |  |
| Acute cancer treatment-related symptoms / physical problems (lasting < 3 months) | 0% | 11% | 15% | 28% | 45% | 0% | | 6% | 15% | 26% | 53% |  |  |  |  |  |  | 0.706** | <0.001 |
|  | 11% | | 15% | 73% | | 6% | | | 15% | 79% | |  |  |  |  |  |  |  |  |
| Acute symptoms / physical problems related to non-cancer causes (lasting < 3 months) | 4% | 8% | 19% | 36% | 34% | 4% | | 4% | 19% | 42% | 32% |  |  |  |  |  |  | 0.787** | <0.001 |
|  | 12% | | 19% | 70% | | 8% | | | 19% | 74% | |  |  |  |  |  |  |  |  |
| Chronic cancer-related symptoms / physical problems (lasting ≥ 3 months) | 0% | 0% | 8% | 26% | 66% | 0% | | 0% | 4% | 21% | 75% |  |  |  |  |  |  | 0.381** | 0.05 |
|  | 0% | | 8% | 92% | | 0% | | | 4% | 96% | |  |  |  |  |  |  |  |  |
| Chronic cancer treatment-related symptoms / physical problems (lasting ≥ 3 months) | 0% | 2% | 11% | 34% | 53% | 0% | | 2% | 6% | 25% | 68% |  |  |  |  |  |  | 0.485** | <0.001 |
|  | 2% | | 11% | 87% | | 2% | | | 6% | 93% | |  |  |  |  |  |  |  |  |
| Chronic symptoms / physical problems related to non-cancer causes (lasting ≥ 3 months) | 2% | 4% | 21% | 32% | 42% | 2% | | 4% | 13% | 36% | 45% |  |  |  |  |  |  | 0.679** | <0.001 |
|  | 6% | | 21% | 74% | | 6% | | | 13% | 81% | |  |  |  |  |  |  |  |  |
| **SPCS generally have the knowledge and skills to manage the following:** | | | | | | | | | | | | | | | | | | | |
| ﻿Acute cancer-related symptoms / physical problems (lasting < 3 months) | 0% | 8% | 9% | 28% | 55% | 0% | | 4% | 9% | 15% | 72% |  | | | | | | 0.739** | <0.001 |
|  | 8% | | 9% | 83% | | 4% | | | 9% | 87% | |  |  |  |  |  |  |  |  |
| Acute cancer treatment-related symptoms / physical problems (lasting < 3 months) | 2% | 13% | 11% | 34% | 40% | 2% | | 8% | 11% | 26% | 53% |  |  |  |  |  |  | 0.816** | <0.001 |
|  | 15% | | 11% | 74% | | 10% | | | 11% | 97% | |  |  |  |  |  |  |  |  |
| Acute symptoms / physical problems related to non-cancer causes (lasting < 3 months) | 2% | 9% | 21% | 36% | 32% | 2% | | 4% | 23% | 42% | 30% |  |  |  |  |  |  | 0.947** | <0.001 |
|  | 11% | | 21% | 68% | | 6% | | | 23% | 72% | |  |  |  |  |  |  |  |  |
| Chronic cancer-related symptoms / physical problems (lasting ≥ 3 months) | 0% | 2% | 11% | 28% | 58% | 0% | | 2% | 4% | 19% | 75% |  |  |  |  |  |  | 0.671** | <0.001 |
|  | 2% | | 11% | 86% | | 2% | | | 4% | 96% | |  |  |  |  |  |  |  |  |
| Chronic cancer treatment-related symptoms / physical problems (lasting ≥ 3 months) | 2% | 4% | 13% | 43% | 38% | 2% | | 4% | 8% | 43% | 43% |  |  |  |  |  |  | 0.879** | <0.001 |
|  | 6% | | 13% | 81% | | 6% | | | 8% | 86% | |  |  |  |  |  |  |  |  |
| Chronic symptoms / physical problems related to non-cancer causes (lasting ≥ 3 months) | 4% | 9% | 21% | 42% | 25% | 4% | | 8% | 15% | 55% | 19% |  |  |  |  |  |  | 0.903** | <0.001 |
|  | 13% | | 21% | 67% | | 12% | | | 15% | 74% | |  |  |  |  |  |  |  |  |
| **SPCS services should have more training in the following areas:** | | | | | | | | | | | | | | | | | | | |
| Assessment of acute side effects of anticancer treatment (especially novel anticancer treatments) |  | | | | | 10% | | 8% | 10% | 33% | 40% | 8% | | 4% | 10% | 22% | 55% | 0.892** | <0.001 |
|  |  |  |  |  |  | 18% | | | 10% | 73% | | 12% | | | 10% | 77% | |  |  |
| Management of acute side effects of anticancer treatment (especially novel anticancer treatments) |  |  |  |  |  | 10% | | 6% | 10% | 35% | 40% | 8% | | 0% | 8% | 33% | 51% | 0.902** | <0.001 |
|  |  |  |  |  |  | 16% | | | 10% | 75% | | 8% | | | 8% | 84% | |  |  |
| Assessment of chronic / long-term effects of anticancer treatment (especially novel anticancer treatments) |  |  |  |  |  | 2% | | 10% | 10% | 33% | 46% | 2% | | 4% | 8% | 20% | 65% | 0.826** | <0.001 |
|  |  |  |  |  |  | 12% | | | 10% | 79% | | 6% | | | 8% | 85% | |  |  |
| Management of chronic / long-term effects of anticancer treatment (especially novel anticancer treatments) |  |  |  |  |  | 4% | | 10% | 10% | 29% | 48% | 4% | | 4% | 6% | 27% | 59% | 0.873** | <0.001 |
|  |  |  |  |  |  | 14% | | | 10% | 77% | | 8% | | | 6% | 86% | |  |  |
| Assessment of late / delayed effects of anticancer treatment (especially novel anticancer treatments) |  |  |  |  |  | 6% | | 13% | 8% | 33% | 40% | 4% | | 10% | 6% | 22% | 57% | 0.870** | <0.001 |
|  |  |  |  |  |  | 19% | | | 8% | 73% | | 14% | | | 6% | 79% | |  |  |
| Management of late / delayed effects of anticancer treatment (especially novel anticancer treatments) |  |  |  |  |  | 4% | | 13% | 8% | 35% | 40% | 2% | | 10% | 6% | 29% | 53% | 0.890** | <0.001 |
|  |  |  |  |  |  | 17% | | | 8% | 75% | | 12% | | | 6% | 82% | |  |  |

| **Key** |  |  |  |  |
| --- | --- | --- | --- | --- |
| SD | Strongly disagree | | rho | Spearman’s rank correlation coefficient between relevant subsequent rounds |
| MD | Moderately disagree | | * | Correlation is significant at the 0.05 level (2-tailed) |
| N | Neither agree nor disagree | | ** | Correlation is significant at the 0.01 level (2-tailed) |
| MA | Moderately agree | | Shaded box | Consensus reached (≥75%) |
| SA | Strongly agree | |  |  |
| SPCS | Specialist palliative care | |  |  |
| SPCS | Specialist palliative care services | | |  |
|  |  |  |  |  |

S2b. Extended role of Specialist Palliative Care for individuals with a history of cancer who have completed anticancer treatment and have no evidence of disease – ONC group results

| **Statement** | **Round 1** | | | | | **Round 2** | | | | | | **Round 3** | | | | | **rho** | **p value** |
| --- | --- | --- | --- | --- | --- | --- | --- | --- | --- | --- | --- | --- | --- | --- | --- | --- | --- | --- |
|  | **SD** | **MD** | **N** | **MA** | **SA** | **SD** | | **MD** | **N** | **MA** | **SA** | **SD** | **MD** | **N** | **MA** | **SA** |  |  |
| **Section 4: Extended role of Specialist Palliative Care for individuals with a history of cancer who have completed anticancer treatment and have no evidence of disease** | | | | | | | | | | | | | | | | | | |
| **SPCS should have a role in supporting the following components of care** | | | | | | | | | | | | | | | | | | |
| ﻿Prevention of recurrent and new cancers | 42% | 30% | 9% | 11% | 8% |  | | | | | | | | | | | | |
|  | 72% | | 9% | 19% | |  |  |  |  |  |  |  |  |  |  |  |  |  |
| ﻿Prevention of other late effects | 28% | 23% | 9% | 26% | 13% |  |  |  |  |  |  |  |  |  |  |  |  |  |
|  | 51% | | 9% | 39% | |  |  |  |  |  |  |  |  |  |  |  |  |  |
| ﻿Surveillance for cancer spread, recurrence, or second cancers | 45% | 30% | 9% | 8% | 8% |  |  |  |  |  |  |  |  |  |  |  |  |  |
|  | 75% | | 9% | 16% | |  |  |  |  |  |  |  |  |  |  |  |  |  |
| ﻿Surveillance of physical late effects | 26% | 21% | 6% | 30% | 17% |  |  |  |  |  |  |  |  |  |  |  |  |  |
|  | 47% | | 6% | 47% | |  |  |  |  |  |  |  |  |  |  |  |  |  |
| ﻿Surveillance of psychosocial late effects | 25% | 21% | 9% | 28% | 17% |  |  |  |  |  |  |  |  |  |  |  |  |  |
|  | 46% | | 9% | 45% | |  |  |  |  |  |  |  |  |  |  |  |  |  |
| ﻿Intervention for pain as a late effect | 13% | 6% | 8% | 43% | 30% |  |  |  |  |  |  |  |  |  |  |  |  |  |
|  | 19% | | 8% | 73% | |  |  |  |  |  |  |  |  |  |  |  |  |  |
| ﻿Intervention for other physical late effects | 13% | 11% | 13% | 36% | 26% |  |  |  |  |  |  |  |  |  |  |  |  |  |
|  | 24% | | 13% | 62% | |  |  |  |  |  |  |  |  |  |  |  |  |  |
| ﻿Intervention for psychosocial late effects | 15% | 8% | 17% | 36% | 25% |  |  |  |  |  |  |  |  |  |  |  |  |  |
|  | 23% | | 17% | 61% | |  |  |  |  |  |  |  |  |  |  |  |  |  |
| ﻿Managing “fear of cancer recurrence” | 23% | 25% | 8% | 32% | 13% |  |  |  |  |  |  |  |  |  |  |  |  |  |
|  | 48% | | 8% | 45% | |  |  |  |  |  |  |  |  |  |  |  |  |  |
| ﻿Rehabilitation | 23% | 21% | 13% | 30% | 13% |  |  |  |  |  |  |  |  |  |  |  |  |  |
|  | 44% | | 13% | 43% | |  |  |  |  |  |  |  |  |  |  |  |  |  |
| ﻿Supporting occupational issues / return to work | 26% | 26% | 8% | 26% | 13% |  |  |  |  |  |  |  |  |  |  |  |  |  |
|  | 52% | | 8% | 39% | |  |  |  |  |  |  |  |  |  |  |  |  |  |
| ﻿Managing financial problems (“financial toxicity”) | 28% | 30% | 11% | 25% | 6% |  |  |  |  |  |  |  |  |  |  |  |  |  |
|  | 58% | | 11% | 31% | |  |  |  |  |  |  |  |  |  |  |  |  |  |
| ﻿Supporting concerns related to insurance | 36% | 25% | 9% | 26% | 4% |  |  |  |  |  |  |  |  |  |  |  |  |  |
|  | 61% | | 9% | 30% | |  |  |  |  |  |  |  |  |  |  |  |  |  |
| ﻿Supporting caregivers | 19% | 19% | 13% | 32% | 17% |  |  |  |  |  |  |  |  |  |  |  |  |  |
|  | 38% | | 13% | 49% | |  |  |  |  |  |  |  |  |  |  |  |  |  |
| ﻿Supporting coordination between specialists and primary care providers | 19% | 25% | 8% | 32% | 17% |  |  |  |  |  |  |  |  |  |  |  |  |  |
|  | 44% | | 8% | 49% | |  |  |  |  |  |  |  |  |  |  |  |  |  |
| Prevention of second cancers |  | | | | | 54% | | 25% | 4% | 10% | 8% | 65% | 22% | 0% | 6% | 6% | 0.837** | <0.001 |
|  |  |  |  |  |  | 79% | | | 4% | 18% | | 87% | | 0% | 12% | |  |  |
| ﻿Surveillance for recurrence or second cancers |  |  |  |  |  | 52% | | 31% | 8% | 2% | 8% | 63% | 27% | 6% | 0% | 4% | 0.802** | <0.001 |
|  |  |  |  |  |  | 83% | | | 8% | 10% | | 90% | | 6% | 4% | |  |  |
| ﻿Surveillance of physical "late effects" |  |  |  |  |  | 29% | | 21% | 2% | 35% | 13% | 37% | 10% | 0% | 43% | 10% | 0.947** | <0.001 |
|  |  |  |  |  |  | 50% | | | 2% | 48% | | 47% | | 0% | 53% | |  |  |
| ﻿Surveillance of psychological distress |  |  |  |  |  | 29% | | 17% | 6% | 35% | 13% | 33% | 8% | 0% | 53% | 6% | 0.876** | <0.001 |
|  |  |  |  |  |  | 46% | | | 6% | 48% | | 41% | | 0% | 59% | |  |  |
| Management of pain |  |  |  |  |  | 13% | | 4% | 8% | 50% | 25% | 10% | 4% | 4% | 59% | 22% | 0.839** | <0.001 |
|  |  |  |  |  |  | 17% | | | 8% | 75% | | 14% | | 4% | 81% | |  |  |
| Management of other physical symptoms / problems |  |  |  |  |  | 13% | | 8% | 13% | 42% | 23% | 12% | 6% | 6% | 55% | 20% | 0.880** | <0.001 |
|  |  |  |  |  |  | 21% | | | 13% | 65% | | 18% | | 6% | 75% | |  |  |
| Management of psychological distress |  |  |  |  |  | 15% | | 10% | 12% | 44% | 19% | 18% | 8% | 6% | 55% | 12% | 0.856** | <0.001 |
|  |  |  |  |  |  | 25% | | | 12% | 63% | | 26% | | 6% | 67% | |  |  |
| ﻿Managing “fear of cancer recurrence” |  |  |  |  |  | 23% | | 27% | 6% | 33% | 12% | 27% | 24% | 4% | 37% | 8% | 0.977** | <0.001 |
|  |  |  |  |  |  | 50% | | | 6% | 45% | | 51% | | 4% | 45% | |  |  |
| Rehabilitation |  |  |  |  |  | 23% | | 21% | 12% | 31% | 13% | 29% | 16% | 12% | 37% | 6% | 0.952** | <0.001 |
|  |  |  |  |  |  | 44% | | | 12% | 44% | | 45% | | 12% | 43% | |  |  |
| ﻿Supporting occupational issues / return to work |  |  |  |  |  | 27% | | 29% | 6% | 27% | 12% | 33% | 29% | 4% | 29% | 6% | 0.922** | <0.001 |
|  |  |  |  |  |  | 56% | | | 6% | 39% | | 62% | | 4% | 35% | |  |  |
| Managing financial problems (“financial toxicity”) |  |  |  |  |  | 31% | | 33% | 6% | 25% | 6% | 35% | 33% | 6% | 22% | 4% | 0.932** | <0.001 |
|  |  |  |  |  |  | 64% | | | 6% | 31% | | 68% | | 6% | 26% | |  |  |
| ﻿Supporting caregivers |  |  |  |  |  | 19% | | 21% | 10% | 35% | 15% | 24% | 12% | 8% | 47% | 8% | 0.880** | <0.001 |
|  |  |  |  |  |  | 40% | | | 10% | 50% | | 36% | | 8% | 55% | |  |  |
| ﻿Coordination between specialists and primary care providers |  |  |  |  |  | 19% | | 25% | 4% | 35% | 17% | 24% | 18% | 0% | 43% | 14% | 0.875** | <0.001 |
|  |  |  |  |  |  | 44% | | | 4% | 53% | | 42% | | 0% | 57% | |  |  |
| Developing "survivorship care plans" |  |  |  |  |  | 25% | | 19% | 17% | 27% | 12% | 37% | 12% | 8% | 35% | 8% | 0.937** | <0.001 |
|  |  |  |  |  |  | 44% | | | 17% | 39% | | 49% | | 8% | 43% | |  |  |
| **SPCS generally have the knowledge and skills to support the following components of care:** | | | | | | | | | | | | | | | | | | |
| ﻿Prevention of recurrent and new cancers | 30% | 34% | 19% | 17% | 0% |  | | | | | | | | | | | | |
|  | 64% | | 19% | 17% | |  |  |  |  |  |  |  |  |  |  |  |  |  |
| ﻿Prevention of other late effects | 13% | 40% | 17% | 23% | 8% |  |  |  |  |  |  |  |  |  |  |  |  |  |
|  | 53% | | 17% | 31% | |  |  |  |  |  |  |  |  |  |  |  |  |  |
| ﻿Surveillance for cancer spread, recurrence, or second cancers | 32% | 34% | 21% | 11% | 2% |  |  |  |  |  |  |  |  |  |  |  |  |  |
|  | 66% | | 21% | 13% | |  |  |  |  |  |  |  |  |  |  |  |  |  |
| ﻿Surveillance of physical late effects | 11% | 25% | 21% | 28% | 15% |  |  |  |  |  |  |  |  |  |  |  |  |  |
|  | 36% | | 21% | 43% | |  |  |  |  |  |  |  |  |  |  |  |  |  |
| ﻿Surveillance of psychosocial late effects | 9% | 23% | 21% | 34% | 13% |  |  |  |  |  |  |  |  |  |  |  |  |  |
|  | 32% | | 21% | 47% | |  |  |  |  |  |  |  |  |  |  |  |  |  |
| ﻿Intervention for pain as a late effect | 8% | 6% | 11% | 43% | 32% |  |  |  |  |  |  |  |  |  |  |  |  |  |
|  | 14% | | 11% | 75% | |  |  |  |  |  |  |  |  |  |  |  |  |  |
| ﻿Intervention for other physical late effects | 6% | 15% | 17% | 36% | 26% |  |  |  |  |  |  |  |  |  |  |  |  |  |
|  | 21% | | 17% | 62% | |  |  |  |  |  |  |  |  |  |  |  |  |  |
| ﻿Intervention for psychosocial late effects | 8% | 13% | 17% | 45% | 17% |  |  |  |  |  |  |  |  |  |  |  |  |  |
|  | 21% | | 17% | 62% | |  |  |  |  |  |  |  |  |  |  |  |  |  |
| ﻿Managing “fear of cancer recurrence” | 17% | 19% | 19% | 36% | 9% |  |  |  |  |  |  |  |  |  |  |  |  |  |
|  | 36% | | 19% | 45% | |  |  |  |  |  |  |  |  |  |  |  |  |  |
| ﻿Rehabilitation | 23% | 19% | 25% | 26% | 8% |  |  |  |  |  |  |  |  |  |  |  |  |  |
|  | 42% | | 25% | 34% | |  |  |  |  |  |  |  |  |  |  |  |  |  |
| ﻿Supporting occupational issues / return to work | 23% | 25% | 21% | 25% | 8% |  |  |  |  |  |  |  |  |  |  |  |  |  |
|  | 48% | | 21% | 33% | |  |  |  |  |  |  |  |  |  |  |  |  |  |
| ﻿Managing financial problems (“financial toxicity”) | 28% | 19% | 28% | 19% | 6% |  |  |  |  |  |  |  |  |  |  |  |  |  |
|  | 47% | | 28% | 25% | |  |  |  |  |  |  |  |  |  |  |  |  |  |
| ﻿Supporting concerns related to insurance | 28% | 19% | 34% | 13% | 6% |  |  |  |  |  |  |  |  |  |  |  |  |  |
|  | 47% | | 34% | 19% | |  |  |  |  |  |  |  |  |  |  |  |  |  |
| ﻿Supporting caregivers | 15% | 15% | 9% | 34% | 26% |  |  |  |  |  |  |  |  |  |  |  |  |  |
|  | 30% | | 9% | 60% | |  |  |  |  |  |  |  |  |  |  |  |  |  |
| ﻿Supporting coordination between specialists and primary care providers | 13% | 15% | 21% | 32% | 19% |  |  |  |  |  |  |  |  |  |  |  |  |  |
|  | 28% | | 21% | 51% | |  |  |  |  |  |  |  |  |  |  |  |  |  |
| Prevention of second cancers |  | | | | | 35% | | 42% | 15% | 8% | 0% | 37% | 55% | 8% | 0% | 0% | 0.725** | <0.001 |
|  |  |  |  |  |  | 77% | | | 15% | 8% | | 92% | | 8% | 0% | |  |  |
| ﻿Surveillance for recurrence or second cancers |  |  |  |  |  | 35% | | 40% | 17% | 6% | 2% | 41% | 47% | 10% | 2% | 0% | 0.670** | <0.001 |
|  |  |  |  |  |  | 75% | | | 17% | 8% | | 88% | | 10% | 2% | |  |  |
| ﻿Surveillance of physical "late effects" |  |  |  |  |  | 10% | | 31% | 17% | 37% | 6% | 4% | 39% | 18% | 35% | 4% | 0.739** | <0.001 |
|  |  |  |  |  |  | 41% | | | 17% | 43% | | 43% | | 18% | 39% | |  |  |
| ﻿Surveillance of psychological distress |  |  |  |  |  | 8% | | 23% | 19% | 42% | 8% | 4% | 24% | 10% | 55% | 6% | 0.872** | <0.001 |
|  |  |  |  |  |  | 31% | | | 19% | 50% | | 28% | | 10% | 61% | |  |  |
| Management of pain |  |  |  |  |  | 6% | | 4% | 10% | 58% | 23% | 2% | 0% | 6% | 76% | 16% | 0.729** | <0.001 |
|  |  |  |  |  |  | 10% | | | 10% | 81% | | 2% | | 6% | 92% | |  |  |
| Management of other physical symptoms / problems |  |  |  |  |  | 6% | | 13% | 13% | 50% | 17% | 4% | 6% | 6% | 76% | 8% | 0.688** | <0.001 |
|  |  |  |  |  |  | 19% | | | 13% | 67% | | 10% | | 6% | 84% | |  |  |
| Management of psychological distress |  |  |  |  |  | 6% | | 12% | 17% | 56% | 10% | 2% | 10% | 6% | 73% | 8% | 0.770** | <0.001 |
|  |  |  |  |  |  | 18% | | | 17% | 66% | | 12% | | 6% | 81% | |  |  |
| ﻿Managing “fear of cancer recurrence” |  |  |  |  |  | 17% | | 21% | 13% | 42% | 6% | 16% | 10% | 14% | 57% | 2% | 0.845** | <0.001 |
|  |  |  |  |  |  | 39% | | | 13% | 48% | | 26% | | 14% | 59% | |  |  |
| Rehabilitation |  |  |  |  |  | 25% | | 19% | 23% | 27% | 6% | 29% | 10% | 29% | 29% | 4% | 0.959** | <0.001 |
|  |  |  |  |  |  | 44% | | | 23% | 33% | | 39% | | 29% | 33% | |  |  |
| ﻿Supporting occupational issues / return to work |  |  |  |  |  | 27% | | 23% | 21% | 25% | 4% | 33% | 20% | 20% | 22% | 4% | 0.880** | <0.001 |
|  |  |  |  |  |  | 50% | | | 21% | 29% | | 53% | | 20% | 26% | |  |  |
| Managing financial problems (“financial toxicity”) |  |  |  |  |  | 31% | | 17% | 35% | 15% | 2% | 33% | 14% | 37% | 14% | 2% | 0.951** | <0.001 |
|  |  |  |  |  |  | 48% | | | 35% | 17% | | 47% | | 37% | 16% | |  |  |
| ﻿Supporting caregivers |  |  |  |  |  | 15% | | 15% | 8% | 42% | 19% | 8% | 16% | 6% | 55% | 14% | 0.869** | <0.001 |
|  |  |  |  |  |  | 30% | | | 8% | 61% | | 24% | | 6% | 69% | |  |  |
| ﻿Coordination between specialists and primary care providers |  |  |  |  |  | 12% | | 19% | 17% | 38% | 13% | 6% | 18% | 14% | 53% | 8% | 0.845** | <0.001 |
|  |  |  |  |  |  | 31% | | | 17% | 51% | | 24% | | 14% | 61% | |  |  |
| Developing "survivorship care plans" |  |  |  |  |  | 19% | | 29% | 21% | 23% | 8% | 18% | 33% | 18% | 24% | 6% | 0.877** | <0.001 |
|  |  |  |  |  |  | 48% | | | 21% | 31% | | 51% | | 18% | 30% | |  |  |
| **Barriers to extending the input of SPCS to this group:** | | | | | | | | | | | | | | | | | | |
| Financial resources | 6% | 9% | 21% | 25% | 40% | 2% | | 8% | 19% | 21% | 50% |  | | | | | 0.877** | <0.001 |
|  | 15% | | 21% | 65% | | 10% | | | 19% | 71% | |  |  |  |  |  |  |  |
| Human resources | 4% | 6% | 15% | 28% | 47% | 0% | | 4% | 15% | 23% | 58% |  |  |  |  |  | 0.823** | <0.001 |
|  | 10% | | 15% | 75% | | 4% | | | 15% | 81% | |  |  |  |  |  |  |  |
| Other resources | 4% | 8% | 36% | 25% | 28% |  | |  |  |  |  |  |  |  |  |  |  |  |
|  | 12% | | 36% | 53% | |  | |  |  |  |  |  |  |  |  |  |  |  |
| Lack of relevant education and training (e.g. management of chronic cancer treatment-related symptoms / problems) | 8% | 15% | 21% | 28% | 28% | 2% | | 13% | 23% | 29% | 33% |  |  |  |  |  | 0.853** | <0.001 |
|  | 23% | | 21% | 56% | | 15% | | | 23% | 62% | |  |  |  |  |  |  |  |
| Lack of willingness from palliative care professionals | 2% | 19% | 32% | 28% | 19% | 0% | | 17% | 35% | 35% | 13% |  |  |  |  |  | 0.870** | <0.001 |
|  | 21% | | 32% | 47% | | 17% | | | 33% | 48% | |  |  |  |  |  |  |  |
| Lack of willingness from oncology professionals | 9% | 8% | 19% | 43% | 21% | 8% | | 6% | 19% | 52% | 15% |  |  |  |  |  | 0.876** | <0.001 |
|  | 17% | | 19% | 64% | | 14% | | | 19% | 67% | |  |  |  |  |  |  |  |
| Lack of willingness from general practitioners | 9% | 6% | 28% | 42% | 15% | 8% | | 6% | 29% | 44% | 13% |  |  |  |  |  | 0.919** | <0.001 |
|  | 15% | | 28% | 57% | | 14% | | | 29% | 57% | |  |  |  |  |  |  |  |
| Lack of willingness from patients and families | 9% | 17% | 30% | 26% | 17% | 8% | | 15% | 35% | 29% | 13% |  |  |  |  |  | 0.971** | <0.001 |
|  | 26% | | 30% | 43% | | 23% | | | 35% | 42% | |  |  |  |  |  |  |  |
| Misperceptions about the nature of palliative care | 4% | 2% | 23% | 38% | 34% | 2% | | 2% | 21% | 38% | 37% |  |  |  |  |  | 0.918** | <0.001 |
|  | 6% | | 23% | 72% | | 4% | | | 21% | 75% | |  |  |  |  |  |  |  |
| Time resources |  | | | | | 0% | 4% | | 15% | 31% | 50% | 0% | 4% | 10% | 16% | 69% | 0.807** | <0.001 |
|  |  |  |  |  |  | 4% | | | 15% | 81% | | 4% | | 10% | 85% | |  |  |
| **Type of service** | | | | | | | | | | | | | | | | | | |
| A dedicated multidisciplinary specialist team is required |  | | | | | 6% | | 6% | 6% | 35% | 48% | 8% | 2% | 2% | 24% | 63% | 0.767** | <0.001 |
|  |  |  |  |  |  | 12% | | | 6% | 83% | | 10% | | 2% | 87% | |  |  |
| Specialist palliative care services should lead this team |  |  |  |  |  | 35% | | 35% | 17% | 12% | 2% | 39% | 41% | 10% | 8% | 2% | 0.844** | <0.001 |
|  |  |  |  |  |  | 70% | | | 17% | 14% | | 80% | | 10% | 10% | |  |  |
| Specialist palliative care services should be a core member of this team i.e. routinely involved in care |  |  |  |  |  | 10% | | 31% | 13% | 29% | 17% | 8% | 41% | 8% | 31% | 12% | 0.922** | <0.001 |
|  |  |  |  |  |  | 41% | | | 13% | 46% | | 49% | | 8% | 43% | |  |  |
| Specialist palliative care services should be an extended member of this team i.e. support with relevant problems / issues |  |  |  |  |  | 2% | | 10% | 12% | 52% | 25% | 2% | 0% | 10% | 76% | 12% | 0.713** | <0.001 |
|  |  |  |  |  |  | 12% | | | 12% | 77% | | 2% | | 10% | 88% | |  |  |
| Specialist palliative care services should not contribute to this team |  |  |  |  |  | 54% | | 17% | 8% | 17% | 4% | 65% | 18% | 6% | 10% | 0% | 0.808** | <0.001 |
|  |  |  |  |  |  | 71% | | | 8% | 31% | | 83% | | 6% | 10% | |  |  |

| **Key** |  |  |  |  |
| --- | --- | --- | --- | --- |
| SD | Strongly disagree | | rho | Spearman’s rank correlation coefficient between relevant subsequent rounds |
| MD | Moderately disagree | | * | Correlation is significant at the 0.05 level (2-tailed) |
| N | Neither agree nor disagree | | ** | Correlation is significant at the 0.01 level (2-tailed) |
| MA | Moderately agree | | Shaded box | Consensus reached (≥75%) |
| SA | Strongly agree | |  |  |
| SPC | Specialist palliative care | |  |  |
| SPCS | Specialist palliative care services | | |  |
|  |  |  |  |  |

# Supplementary Table S3: Comparison of levels of agreement between SPC and ONC groups regarding final statements

| **Statement** | **x^2^(1)** | **p value** | **n** |
| --- | --- | --- | --- |
| **Section: Definition and core functions of Specialist Palliative Care (SPC)** | | | |
| **Core functions of SPCS** | | | |
| ﻿Management of pain | 0.082 | 0.775 | 53 |
| Management of other physical symptoms | 0.353 | 0.553 | 53 |
| ﻿﻿Management of psychological problems | 0.353 | 0.553 | 53 |
| Emotional support (patient) | 0.489 | 0.484 | 53 |
| Emotional support (family) | 0.144 | 0.704 | 53 |
| Spiritual support | 4.484 | ***0.034*** | 53 |
| Social assistance | 15.684 | ***<0.001*** | 53 |
| Co-ordination of care | 2.883 | 0.090 | 53 |
| Advance care planning | 0.489 | 0.484 | 53 |
| End-of-life care | N/A | | |
| Bereavement care | 7.109 | ***0.008*** | 53 |
| Rehabilitation | 4.479 | ***0.034*** | 53 |
| Religious support | 7.837 | ***0.005*** | 49 |
| Research | 0.726 | 0.394 | 49 |
| Education (other healthcare professionals) | N/A | | |
|  | | | |
| **SPCS should manage the following types of pain:** | | | |
| Acute cancer-related pain (pain for < 3 months) | 0.234 | 0.629 | 53 |
| Acute cancer treatment-related pain (pain for < 3 months) | 2.211 | 0.137 | 53 |
| Acute pain related to non-cancer causes (pain for < 3 months) | 3.965 | ***0.046*** | 53 |
| Chronic cancer-related pain (pain for≥3 months) | 1.017 | 0.313 | 53 |
| Chronic cancer treatment-related pain (pain for≥3 months) | 4.355 | ***0.037*** | 53 |
| Chronic pain related to non-cancer causes (pain for≥3 months) | 0.997 | 0.318 | 53 |
| Individuals with pain and a previous history of opioid misuse / abuse / addiction | 0.503 | 0.478 | 49 |
| Individuals with pain and ongoing problems of opioid misuse / abuse / addiction | 1.503 | 0.220 | 49 |
| Chronic cancer / cancer treatment-related pain – in collaboration with the multidisciplinary chronic pain team | 0.503 | 0.478 | 49 |
| Individuals with cancer / cancer treatment–related pain and a previous history of opioid misuse / abuse / addiction | 1.503 | 0.220 | 49 |
| Individuals with cancer / cancer treatment–related pain and ongoing problems of opioid misuse / abuse / addiction | 1.907 | 0.167 | 49 |
| Individuals with chronic cancer / cancer treatment–related pain and a previous history of opioid misuse / abuse / addiction – in collaboration with the multidisciplinary chronic pain team | 0.058 | 0.810 | 49 |
| Individuals with chronic cancer / cancer treatment–related pain and ongoing problems of opioid misuse / abuse / addiction – in collaboration with the multidisciplinary chronic pain team | 1.306 | 0.253 | 49 |
| Individuals with cancer / cancer treatment–related pain and a previous history of opioid misuse / abuse / addiction – in collaboration with addiction services | 0.134 | 0.714 | 49 |
| Individuals with cancer / cancer treatment–related pain and ongoing problems of opioid misuse / abuse / addiction – in collaboration with addiction services | 1.355 | 0.244 | 49 |
|  | | | |
| **SPCS generally have the knowledge and skills to manage the following:** | | | |
| Acute cancer-related pain (pain for < 3 months) | 8.894 | ***0.003*** | 53 |
| Acute cancer treatment-related pain (pain for < 3 months) | 0.066 | 0.798 | 53 |
| Acute pain related to non-cancer causes (pain for < 3 months) | 10.193 | ***0.001*** | 53 |
| Chronic cancer-related pain (pain for≥3 months) | N/A | | |
| Chronic cancer treatment-related pain (pain for≥3 months) | 4.355 | ***0.037*** | 53 |
| Chronic pain related to non-cancer causes (pain for≥3 months) | 5.499 | ***0.019*** | 53 |
| Individuals with cancer / cancer treatment–related pain and a previous history of opioid misuse / abuse / addiction | 3.029 | 0.082 | 49 |
| Individuals with cancer / cancer treatment–related pain and ongoing problems of opioid misuse / abuse / addiction | 7.537 | ***0.006*** | 49 |
|  | | | |
| **SPCS services should have more education and / or appropriate training in the following areas:** | | | |
| Interventional pain techniques | 0.240 | 0.877 | 49 |
| Physiotherapy interventions | 15.784 | ***<0.001*** | 49 |
| Occupational therapy interventions | 2.768 | 0.096 | 49 |
| Transcutaneous electrical nerve stimulation (TENS), scrambler therapy | 1.306 | 0.253 | 49 |
| Acupuncture, acupressure | 0.575 | 0.448 | 49 |
| Psychological and behavioural therapies / interventions | 0.122 | 0.727 | 49 |
| Complementary and alternative therapies | 0.347 | 0.556 | 49 |
| Pharmacological interventions in addiction recovery | 2.420 | 0.120 | 49 |
| Psychosocial and behavioural interventions in addiction recovery | 0.044 | 0.834 | 49 |
|  | | | |
| **SPCS should manage the following types of non-pain symptoms / physical problems:** | | | |
| ﻿Acute cancer-related symptoms / physical problems (lasting < 3 months) | 4.475 | ***0.034*** | 53 |
| Acute cancer treatment-related symptoms / physical problems (lasting < 3 months) | 2.309 | 0.129 | 53 |
| Acute symptoms / physical problems related to non-cancer causes (lasting < 3 months) | 0.251 | 0.617 | 53 |
| Chronic cancer-related symptoms / physical problems (lasting ≥ 3 months) | 0.144 | 0.704 | 53 |
| Chronic cancer treatment-related symptoms / physical problems (lasting ≥ 3 months) | 1.110 | 0.292 | 53 |
| Chronic symptoms / physical problems related to non-cancer causes (lasting ≥ 3 months) | 6.611 | ***0.010*** | 53 |
|  | | | |
| **SPCS generally have the knowledge and skills to manage the following:** | | | |
| ﻿Acute cancer-related symptoms / physical problems (lasting < 3 months) | 24.929 | ***<0.001*** | 53 |
| Acute cancer treatment-related symptoms / physical problems (lasting < 3 months) | 19.306 | ***<0.001*** | 53 |
| Acute symptoms / physical problems related to non-cancer causes (lasting < 3 months) | 1.143 | 0.285 | 53 |
| Chronic cancer-related symptoms / physical problems (lasting ≥ 3 months) | 0.066 | 0.798 | 53 |
| Chronic cancer treatment-related symptoms / physical problems (lasting ≥ 3 months) | 0.239 | 0.625 | 53 |
| Chronic symptoms / physical problems related to non-cancer causes (lasting ≥ 3 months) | 0.016 | 0.898 | 53 |
|  | | | |
| **SPCS services should have more training in the following areas:** | | | |
| Assessment of acute side effects of anticancer treatment (especially novel anticancer treatments) | 171.000 | ***<0.001*** | 49 |
| Management of acute side effects of anticancer treatment (especially novel anticancer treatments) | 21.066 | ***<0.001*** | 49 |
| Assessment of chronic / long-term effects of anticancer treatment (especially novel anticancer treatments) | 64.474 | ***<0.001*** | 49 |
| Management of chronic / long-term effects of anticancer treatment (especially novel anticancer treatments) | 14.763 | ***<0.001*** | 49 |
| Assessment of late / delayed effects of anticancer treatment (especially novel anticancer treatments) | 139.592 | ***<0.001*** | 49 |
| Management of late / delayed effects of anticancer treatment (especially novel anticancer treatments) | 28.487 | ***<0.001*** | 49 |
|  | | | |
| **Section: Extended role of Specialist Palliative Care for individuals with a history of cancer who have completed anticancer treatment and have no evidence of disease** | | | |
| **SPCS should have a role in supporting the following components of care** | | | |
| Prevention of second cancers | 9.577 | ***0.002*** | 49 |
| ﻿Surveillance for recurrence or second cancers | 0.825 | 0.364 | 49 |
| ﻿Surveillance of physical "late effects" | 10.828 | ***<0.001*** | 49 |
| ﻿Surveillance of psychological distress | 17.767 | ***<0.001*** | 49 |
| Management of pain | 1.150 | 0.284 | 49 |
| Management of other physical symptoms / problems | 0.223 | 0.637 | 49 |
| Management of psychological distress | 0.360 | 0.548 | 49 |
| ﻿Managing “fear of cancer recurrence” | 0.291 | 0.589 | 49 |
| Rehabilitation | 4.744 | ***0.029*** | 49 |
| ﻿Supporting occupational issues / return to work | 14.906 | ***<0.001*** | 49 |
| Managing financial problems (“financial toxicity”) | 11.367 | ***<0.001*** | 49 |
| ﻿Supporting caregivers | 4.656 | ***0.031*** | 49 |
| ﻿Coordination between specialists and primary care providers | 19.246 | ***<0.001*** | 49 |
| Developing "survivorship care plans" | 3.857 | 0.050 | 49 |
|  | | | |
| **SPCS generally have the knowledge and skills to support the following components of care:** | | | |
| Prevention of second cancers | N/A | | |
| ﻿Surveillance for recurrence or second cancers | 4.185 | ***0.041*** | 49 |
| ﻿Surveillance of physical "late effects" | 7.030 | ***0.008*** | 49 |
| ﻿Surveillance of psychological distress | 0.134 | 0.715 | 49 |
| Management of pain | 0.843 | 0.359 | 49 |
| Management of other physical symptoms / problems | 0.266 | 0.606 | 49 |
| Management of psychological distress | 0.026 | 0.873 | 49 |
| ﻿Managing “fear of cancer recurrence” | 0.583 | 0.445 | 49 |
| Rehabilitation | 1.944 | 0.163 | 49 |
| ﻿Supporting occupational issues / return to work | 0.061 | 0.805 | 49 |
| Managing financial problems (“financial toxicity”) | 0.047 | 0.829 | 49 |
| ﻿Supporting caregivers | 1.849 | 0.174 | 49 |
| ﻿Coordination between specialists and primary care providers | 2.393 | 0.122 | 49 |
| Developing "survivorship care plans" | 0.009 | 0.925 | 49 |
|  | | | |
| **Barriers to extending the input of SPCS to this group:** | | | |
| Financial resources | 4.027 | ***0.045*** | 51 |
| Human resources | 5.388 | ***0.020*** | 51 |
| Time resources | 0.019 | 0.892 | 49 |
| Lack of relevant education and training (e.g. management of chronic cancer treatment-related symptoms / problems) | 11.770 | ***<0.001*** | 51 |
| Lack of willingness from palliative care professionals | 0.895 | 0.344 | 51 |
| Lack of willingness from oncology professionals | 1.410 | 0.235 | 51 |
| Lack of willingness from general practitioners | 5.926 | ***0.015*** | 51 |
| Lack of willingness from patients and families | 0.033 | 0.856 | 51 |
| Misperceptions about the nature of palliative care | 0.038 | 0.846 | 51 |
|  | | | |
| **Type of service** | |  |  |
| A dedicated multidisciplinary specialist team is required | 0.045 | 0.832 | 49 |
| Specialist palliative care services should lead this team | 0.058 | 0.810 | 49 |
| Specialist palliative care services should be a core member of this team i.e. routinely involved in care | 0.346 | 0.556 | 49 |
| Specialist palliative care services should be an extended member of this team i.e. support with relevant problems / issues | 4.978 | ***0.026*** | 49 |
| Specialist palliative care services should not contribute to this team | 0.002 | 0.962 | 49 |

Degrees of freedom (DOF)=1 for all statements

For a significance level of 0.05 and one degree of freedom, the critical value is 3.841.

Key:

SPC = specialist palliative care expert group; ONC = oncology expert group

SPCS = specialist palliative care services

x^2^(1) = Chi square test for goodness of fit comparing the levels of agreement of the ONC group with the SPC group for each statement, for one degree of freedom

N/A = unable to perform x^2^ test as at least one group achieved 100% agree / not agree and comparison is not possible

n = number of participants in ONC expert comparison group who responded to the statement

Italicised, bold p value = significant (p<0.05)

Pale grey box = Statement achieved SPC consensus but not ONC consensus

Dark grey box = Statement achieved ONC consensus but not SPC consensus
